# Serotonin Modulation of Dorsoventral Hippocampus in Physiology and Schizophrenia

**DOI:** 10.3390/ijms26157253

**Published:** 2025-07-27

**Authors:** Charalampos L. Kandilakis, Costas Papatheodoropoulos

**Affiliations:** Laboratory of Physiology-Neurophysiology, Department of Medicine, University of Patras, 265 04 Patras, Greece; up1084138@ac.upatras.gr

**Keywords:** serotonin, hippocampus, dorsoventral, dorsal hippocampus, ventral hippocampus, 5-HT receptor, schizophrenia, excitation/inhibition balance, network oscillations, neurodevelopmental disorder, antipsychotics

## Abstract

The serotonergic system, originating in the raphe nuclei, differentially modulates the dorsal and ventral hippocampus, which are implicated in cognition and emotion, respectively. Emerging evidence from rodent models (e.g., neonatal ventral hippocampal lesion, pharmacological NMDA receptor antagonist exposure) and human postmortem studies indicates dorsoventral serotonergic alterations in schizophrenia. These data include elevated 5-HT1A receptor expression in the dorsal hippocampus, linking serotonergic hypofunction to cognitive deficits, and hyperactive 5-HT2A/3 receptor signaling and denser serotonergic innervation in the ventral hippocampus driving local hyperexcitability associated with psychosis and stress responsivity. These dorsoventral serotonergic alterations are shown to disrupt the excitation–inhibition balance, impair synaptic plasticity, and disturb network oscillations, as established by in vivo electrophysiology and functional imaging. Synthesizing these multi-level findings, we propose a novel “dorsoventral serotonin imbalance” model of schizophrenia, in which ventral hyperactivation predominantly contributes to psychotic symptoms and dorsal hypoactivity underlies cognitive deficits. We further highlight promising preclinical evidence that selective targeting of region- and receptor-specific targeting, using both pharmacological agents and emerging delivery technologies, may offer novel therapeutic opportunities enabling symptom-specific strategies in schizophrenia.

## 1. Introduction

The serotonergic system, originating from the raphe nuclei, is a widely distributed and complex neuromodulatory network that regulates a broad range of cellular, circuit-level, cognitive, emotional, and behavioral functions [1,2,3]. Serotonin exerts its effects via seven receptor subtypes (5-HT1R–5-HT7R), which influence neuronal excitability, neurotransmitter release, synaptic plasticity, excitation–inhibition balance, and neural oscillations [4,5,6,7,8]. Serotonergic dysfunction has also been implicated in various psychiatric disorders, including schizophrenia, depression, and autism spectrum disorder [9,10,11].

Schizophrenia is a chronic and severe psychiatric disorder with a lifetime prevalence of approximately 1%, associated with reduced life expectancy [12,13] and substantial impairment in daily functioning [14]. Clinically, it manifests as a heterogeneous constellation of symptoms, including positive symptoms (delusions and hallucinations), negative symptoms (anhedonia, avolition, and asociality), and cognitive deficits (e.g., impairments in attention, episodic memory, and working memory) [15,16].

Components of the serotonergic system are altered in patients with schizophrenia [17,18,19], and atypical antipsychotics exert part of their therapeutic effects via serotonin receptor modulation [20,21]. Moreover, serotonin plays key roles in neurodevelopmental processes implicated in schizophrenia onset [22,23]. Although current treatments are relatively effective for positive symptoms, they show limited efficacy in addressing negative and cognitive symptoms [24,25]. Thus, identifying the underlying pathophysiological mechanisms is essential for developing more effective interventions.

Schizophrenia is associated with functional dysregulation across multiple brain regions, including the hippocampus, which contributes to learning and memory, spatial navigation, cognition, sensorimotor integration, emotional regulation, social behavior, anxiety, and stress responses [26,27,28,29,30,31]. Reductions in hippocampal volume have been consistently observed in patients with psychosis [12], including in individuals at the first episode [32,33]. However, a comprehensive mechanistic understanding of how the hippocampus contributes to the diverse symptom domains of schizophrenia remains elusive.

Despite extensive research on serotonin and hippocampal function, existing reviews have not integrated how region-specific serotonergic modulation along the dorsoventral hippocampal axis contributes to the clinical symptoms of schizophrenia. The current literature is limited by a lack of direct comparison between dorsal (cognitive) and ventral (emotional, psychosis-linked) serotonergic mechanisms, and by minimal focus on how these distinctions inform symptom heterogeneity and targeted therapy. This review addresses these gaps by synthesizing molecular, circuit, and translational data to propose a dorsoventral imbalance model, highlighting how distinct serotonergic alterations in the dorsal versus ventral hippocampus underlie the cognitive, emotional, and psychotic features of schizophrenia, and by suggesting region-specific therapeutic strategies.

Specifically, emerging evidence from both preclinical and clinical studies indicates that the dorsal and ventral hippocampus, corresponding to the posterior and anterior hippocampus in humans, respectively, are differentially involved in the pathophysiology of schizophrenia [34,35,36,37,38]. This pattern is consistent with broader dorsoventral segregation in hippocampal function, connectivity, intrinsic physiology, and susceptibility to pathology [26,27,28,29,30,31,39,40,41,42,43,44,45]. The dorsal hippocampus is primarily involved in cognitive functions such as episodic memory, spatial navigation, attention, and decision-making [46,47,48,49], while the ventral hippocampus is more strongly associated with emotional regulation, motivation, reward-related learning, and stress responsiveness [50,51,52,53]. Furthermore, dorsoventral specialization extends to neuromodulatory influences, including cholinergic, adrenergic, and dopaminergic signaling [40], and growing evidence suggests that serotonergic modulation also differs along the longitudinal axis of the hippocampus, both in physiological function and in psychiatric conditions such as schizophrenia [4,54,55,56]. Additionally, hippocampal serotonergic dysfunction has been increasingly implicated in schizophrenia pathophysiology [57,58,59]. However, how serotonergic modulation along the dorsoventral hippocampal axis contributes to the disorder remains largely unclear.

**Figure 1:**
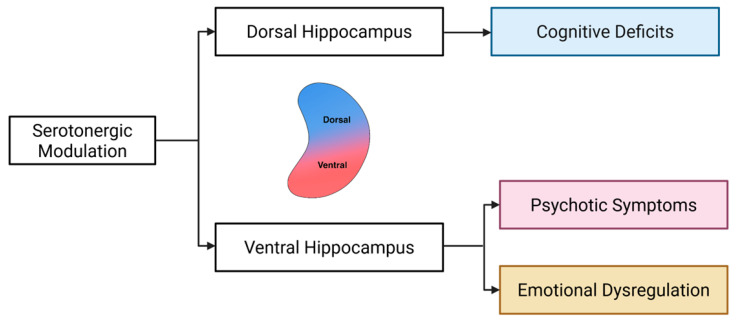
Proposed model of dorsoventral serotonergic imbalance in schizophrenia. Serotonergic modulation impacts the dorsal and ventral hippocampus in functionally distinct ways. Dorsal serotonergic dysfunction is linked to cognitive deficits, while ventral hyperactivation contributes to psychotic symptoms and emotional disturbances. This region-specific imbalance may underlie symptom heterogeneity in schizophrenia.

Building on these insights, we propose a model of dorsoventral serotonergic imbalance in schizophrenia. In this framework, serotonin-driven hyperactivation of the ventral hippocampus contributes to psychotic symptoms [60,61]; persistent dysregulation in this region may underlie emotional disturbances [62,63,64], and impaired serotonergic signaling in the dorsal hippocampus is implicated in cognitive deficits [65,66] (Figure 1). By targeting region-specific serotonin receptors, this model offers a novel perspective on the symptom heterogeneity of schizophrenia and identifies potential avenues for precise, circuit-based therapeutic interventions.

This review was conducted as a narrative literature synthesis informed by systematic search principles. A structured PubMed search was performed up to September 2024 using combinations of keywords such as “serotonin,” “5-HT receptors,” “dorsal hippocampus,” “ventral hippocampus,” “schizophrenia,” “psychosis,” “plasticity,” “neural rhythms,” “development,” and “antipsychotic.” Studies were included if they addressed serotonin receptor function in the hippocampus, reported dorsoventral differences, or explored serotonergic involvement in schizophrenia. Both animal and human research were considered. Additionally, reference lists of key articles were reviewed to identify additional sources.

We begin by outlining general aspects of the hippocampal serotonergic system. This is followed by a discussion on the role of serotonin in hippocampal network dynamics, encompassing synaptic plasticity, oscillatory activity, and neurodevelopmental processes. We then review current evidence for serotonergic dysfunction in schizophrenia, focusing on innervation, receptor expression, and serotonin levels, with emphasis, where available, on dorsoventral distinctions. Subsequently, we examine the effects of atypical antipsychotics on hippocampal circuitry, as well as the region’s potential involvement in the clinical condition of psychosis of epilepsy. Finally, we propose a model of dorsoventral serotonergic dysfunction that accounts for the clinical complexity of schizophrenia to guide the development of region-specific, symptom-oriented therapeutic strategies.

## 2. Organization of the Hippocampal Serotonergic System

The serotonergic system is a widely distributed and complex neuromodulatory system in the brain that mediates a variety of cellular, cognitive, emotional, and behavioral responses. Serotonergic projections originate from the raphe nuclei, located along the midline of the brainstem, where the cell bodies form nine groups of serotonin-containing neurons, namely B1-B9 [67]. The forebrain is innervated by the dorsal (B6 and B7) and the median raphe nucleus (B8 and its extension B9), while B1–B4 groups innervate the brainstem and the spinal cord. Interestingly, the dorsal raphe nuclei (dRNu) and median raphe nuclei (mRNu) differ in their anatomical connections. While the dRNu mainly innervate the prefrontal cortex, the lateral septum, the amygdala, the striatum, and the ventral hippocampus, the mRNu innervate the medial septum, the dorsal hippocampus, the hypothalamus, and parts of the cortex [68]. It is worth mentioning that the serotonergic neurons of the raphe nuclei are also targeted by the other neuromodulatory systems, such as the dopaminergic, cholinergic, and noradrenergic systems, and by cortical and limbic structures [69], hence creating the anatomical prerequisites for fine and complex neuromodulatory interactions.

The modulatory effects of serotonin are mediated by 7 types of membrane receptors (5-HT1-7Rs) and at least 14 5-HTR subtypes with a widespread expression in the brain and distinct pharmacological and physiological properties; for reviews, see [1,2,3,70]. Serotonergic receptors are either somatodendritically located on neurons of the raphe nuclei where they act as inhibitory autoreceptors (5-HT1A), on axon terminals of other neuromodulatory neurons, such as cholinergic, or on glutamatergic and GABAergic cells, where they act as heteroreceptors, excitatory or inhibitory. All 5-HTRs are expressed in the hippocampus [71,72]; for a review, see [73]. With the exception of the 5-HT3R, which is a ligand-gated cation channel permeable to sodium, potassium, and calcium ions, all 5-HTRs are G-protein-coupled receptors (GPCRs) [1,2,3]. These GPCRs are primarily coupled to distinct intracellular signaling cascades: 5-HT1A/BRs and 5-HT5Rs signal via Gi/Go proteins, 5-HT2A/CRs via Gαq, and 5-HT4Rs, 5-HT6Rs, and 5-HT7Rs via Gαs. Each 5-HTR subtype modulates a wide array of downstream molecular targets. 5-HT1A/BRs and 5-HT5Rs influence GIRK and voltage-gated calcium channels, activate the MAPK–ERK pathway, and modulate AMPA and NMDA receptor activity [74,75,76,77,78,79,80,81,82,83,84]. 5-HT2A/CRs engage the PLC–PIP2–DAG signaling cascade, inhibit potassium channels, and interact with postsynaptic scaffolding proteins [85,86,87,88,89,90,91]. 5-HT3Rs are homopentameric or heteropentameric cation channels permeable to Na^+^, K^+^, and Ca^2+^, while they also interact with α4 nicotinic receptor subunits and with IGF-1 pathways [92,93,94,95,96,97]. 5-HT4Rs, 5-HT6Rs, and 5-HT7Rs activate the adenylyl cyclase–cAMP–PKA cascade and influence transcriptional pathways involving CREB and BDNF [98,99,100,101,102,103,104,105,106,107,108,109,110,111,112,113,114,115,116,117,118,119,120,121]. Beyond intracellular signaling, 5-HTRs regulate neurotransmitter release from neuromodulatory axon terminals. Their modulatory effects on serotonin, dopamine, acetylcholine, and norepinephrine release have been extensively described [5,6] and are summarized in Table 1.

### 2.1. Structural Features of the Hippocampal Serotonergic System

As previously mentioned, the serotonergic input of the ventral hippocampus originates from the mRNu and dRNu, while the dorsal hippocampus is solely innervated by the mRNu. The axons sprouting from these raphe nuclei also differ in their morphological and physiological characteristics. More specifically, the axons originating from the dRNu are fine with small varicosities, containing large densities of 5-HT transporters (SERTs), and because of that, they are more sensitive to the neurotoxic effects of MDMA (3,4-methylenedioxymethamphetamine) and PCA (parachloroamphetamine). On the other hand, the axons originating from the mRNu are coarse with large varicosities, largely lacking 5-HT transporters and being more resilient to the toxicity of MDMA and PCP [122,123,124]. These differences may have important implications for the way of transmission, i.e., volume neuromodulatory transmission by fine fibers without large varicosities versus cell-specific modulation of hippocampal cells by beaded fibers with large varicosities. The serotonergic varicosities in the hippocampus are mainly associated with volume transmission [125], while stimulation of median raphe neurons elicits a spatiotemporally precise excitatory response of hippocampal interneurons [126].

The distribution of the serotonergic innervation of the hippocampus has been examined with light microscopy and radioautographic detection in the rat brain [56], and three important features have emerged. Firstly, the serotonergic innervation of the hippocampus follows a layer-specific organization, where the highest innervation is found in the molecular layer of CA1 and the stratum oriens of CA3 (suggesting a preference for the apical and basal dendrites, respectively), moderate innervation in the molecular and polymorph layer of the DG, and minimal innervation in the pyramidal layer of CA regions and the granular cell layer of the DG. Furthermore, serotonergic innervation is denser in CA3 than in the CA1 region. Interestingly, the ventral hippocampus receives greater serotonergic input compared with the dorsal hippocampus [54,56].

The 5-HT1ARs, 5-HT1BRs, 5-HT1FRs, 5-HT2ARs, 5-HT2BRs, 5-HT2CRs, 5-HT3ARs, 5-HT4Rs, 5-HT5ARs, 5-HT5BRs, 5-HT6Rs and 5-HT7Rs are expressed in the hippocampus [71,72]; for a review, see [73]. Recent transcriptomic analyses in rodents reveal high expression of 5-HT1A, 5-HT1F, 5-HT3A, and 5-HT4 receptors in the hippocampus, with moderate expression of 5-HT2A and 5-HT7Rs [127]. More specifically, 5-HT1ARs are strongly expressed in CA1 and with lesser density in the CA3 pyramidal cell layer and in the granule cell layer of the DG [71], often with extrasynaptic localization [128]. 5-HT1BRs are weakly expressed in the pyramidal cell layer of CA3 and CA1 and in the granule cell layer of the DG [129]. 5-HT2ARs are also expressed in these hippocampal regions [130]. 5-HT2CRs are mainly found in the pyramidal cell layer of ventral CA3 region and in stratum oriens and radiatum of the dorsal CA1 region [131], 5-HT3Rs are predominantly expressed in hippocampal inhibitory interneurons [132], 5-HT4Rs are expressed in the pyramidal cell layer of the CA3 region and highly expressed in the granule cell layer of the DG, while 5-HT5Rs are moderately expressed in CA1 pyramidal cells [71]. 5-HT6Rs are found in every hippocampal area [72], and 5-HT7Rs are found in the CA1 and CA3 pyramidal cell layer [98] and in the DG [71]. Interestingly, 5-HTRs are differentially expressed along the dorsoventral axis of the hippocampus. 5-HT1ARs are highly expressed in the dorsal CA1 hippocampal region and in the ventral CA3 and DG regions [71], but see also [133]. Furthermore, 5-HT2CRs are mainly expressed in the stratum oriens and stratum radiatum of the dorsal CA1 region and in the pyramidal cell layer of the ventral CA3 region. Serotonin receptors, expressed by both pyramidal cells and GABAergic interneurons, play key roles in modulating excitability and synaptic plasticity in the hippocampus, thereby influencing network oscillations, as discussed in the following sections. Table 2 provides an overview of the structural and functional characteristics of hippocampal 5-HTRs.

### 2.2. Cellular Mechanisms of Serotonin Receptors in the Hippocampus

The following sections examine the fundamental physiological actions of individual 5-HT receptor subtypes (5-HTRs) on excitatory and inhibitory hippocampal systems, with an emphasis on their potential relevance to schizophrenia pathophysiology. For a more disease-specific discussion, refer to Section 4.1. Particular focus is given to the effects of 5-HTRs on glutamatergic and GABAergic neurons, as these cell types constitute the core elements governing E/I dynamics that are essential for normal hippocampal circuit function.

#### 2.2.1. 5-HT1Rs

In the hippocampus, 5-HT1ARs and 5-HT1BRs modulate the excitability of pyramidal cells and inhibitory interneurons. For instance, application of serotonin in CA1 pyramidal cells produces a 5-HT1AR-mediated biphasic response consisting of a hyperpolarization followed by a longer-lasting depolarization [74]. Exogenously applied serotonin produces a reversible and dose-dependent reduction in the amplitude of the population spike (PS) in vitro [198], and endogenous serotonin tonically inhibits the spontaneous firing of dorsal hippocampus CA1 pyramidal neurons in vivo via 5-HT1ARs [199]. However, elevating endogenous serotonin levels with MDMA reduces population spike (PS) amplitude in the CA1 region of the dorsal hippocampus while increasing it in the ventral hippocampus, presumably via activation of 5-HT1ARs [4]. These findings suggest that serotonin may significantly contribute to shaping the emotional salience of information processed by the ventral hippocampus, which is preferentially involved in emotional regulation. Furthermore, serotonin-releasing drugs enhance the DG granule cells’ response to perforant path stimulation via 5-HT1ARs. Finally, presynaptic 5-HT1BRs inhibit the release of glutamate in the hippocampus [200].

Both 5-HT1ARs and 5-HT1BRs inhibit hippocampal interneurons [201,202], leading to a reduction in both fast and slow inhibitory postsynaptic potentials (IPSPs) in CA1 pyramidal cells [203]. It should be noted that in contrast to the excitatory 5-HT3Rs, which are only expressed in a subset of neurons, all GABAergic boutons in the hippocampus contain 5-HT1ARs [201,204]. Interestingly, serotonin modulates GABAergic transmission in a cell-specific manner [205]. Specifically, activation of 5-HT1BRs, which are primarily expressed in the stratum pyramidale of the CA1 region, suppresses cholecystokinin (CCK)-containing basket cells that receive input from CA1, but not CA3, pyramidal cells. This suppression leads to disinhibition of CA1 pyramidal neurons and an increased integration time window for spike timing [206]. These actions may crucially impact the input/output properties of the CA1 hippocampal circuitry, considering the basic role of CCK neurons in modulating CA1 circuitry [207,208].

Through their regulatory actions on both excitatory and inhibitory transmission, 5-HT1ARs represent a key modulator of E/I balance in both the dorsal and ventral regions of the hippocampus [4,137]. Notably, ventral hippocampal (VH) hyperexcitability is a prominent circuit-level feature associated with the emergence of positive symptoms in schizophrenia [209,210,211]. As detailed in Section 4.1, upregulation of 5-HT1ARs has been reported in both animal models and postmortem human studies. Although such findings are not derived directly from clinical imaging, they nonetheless offer important mechanistic insights into schizophrenia pathophysiology.

Increased 5-HT1AR expression in the VH may enhance local E/I ratio, potentially contributing to psychosis [4,212], while upregulated 5-HT1AR-dependent signaling in the dorsal hippocampus (DH) could lead to excessive inhibition and associated cognitive dysfunction [36,213]. Supporting this, pharmacological blockade of 5-HT1ARs enhances glutamate and acetylcholine release in the hippocampus and is associated with improved cognitive performance [138]. Antagonism of 5-HT1AR also rescues working memory impairments induced by cholinergic suppression [139] and prevents spatial learning deficits resulting from NMDA and AMPA receptor blockade in the hippocampus [140,141]. In addition to 5-HT1ARs, 5-HT1BRs may also contribute to hippocampal circuit dysfunction in schizophrenia. Specifically, by modulating the activity of cholecystokinin-positive (CCK+) interneurons, 5-HT1BRs may influence cognitive processes vulnerable in schizophrenia [207,208]. Disrupted CCK+ interneuron function has been associated with aberrant neural oscillations and impaired cognition, highlighting a potential pathogenic mechanism [214].

#### 2.2.2. 5-HT2Rs

Contrary to the 5-HT1Rs, the cellular and circuitry effects of 5-HT2Rs are less extensively studied. It has been shown that 5-HT2Rs modulate the hippocampal output through the CA1 region by increasing GABAergic synaptic activity [215] and exciting a subclass of inhibitory interneurons located on the border of the stratum radiatum and stratum lacunosum/moleculare [216]. In neocortical slices, low concentrations of the 5-HT2R antagonist and 5-HT reuptake inhibitor trazodone inhibit GABAergic interneurons by antagonizing the excitatory action of 5-HT2A heteroreceptors on axon terminals, while at higher doses, it enhances GABA release by increasing the extracellular concentration of serotonin [217]. In the hippocampus, MDMA increases the extracellular concentration of glutamate via activation of 5-HT2A/2CR [218], while it reduces the activity of parvalbumin-expressing GABAergic cells presumably by the 5-HT2AR-PGE2 signaling pathway [219]. Through these actions, MDMA leads to an excitation–inhibition imbalance that is thought to be crucially involved in the pathophysiology of schizophrenia. Interestingly, schizophrenic patients present reduced density of hippocampal PV-expressing interneurons [220].

5-HT2ARs are strongly implicated in schizophrenia, as their activation constitutes a well-established pharmacological model of psychosis [16], and most second-generation antipsychotic drugs exert therapeutic effects, at least in part, through potent antagonism of 5-HT2ARs [221]. Given the pivotal role of 5-HT2AR signaling in psychosis, future studies should explore the dorsoventral-specific contributions of these receptors to E/I balance, both under physiological conditions and in the context of schizophrenia. Beyond psychotic symptoms, furthermore, activity of 5-HT2A/CRs in the hippocampus has been associated with the regulation of anxiety [144,145], depressive-like behaviors [146], and cognitive functions such as learning and memory [147,148]. Consequently, aberrant 5-HT2AR signaling may also underlie specific emotional and cognitive disturbances observed in patients with schizophrenia. Understanding how 5-HT2AR function differs across segments along the long axis of the hippocampus could therefore advance the development of more targeted therapeutic approaches.

#### 2.2.3. 5-HT3Rs

The 5-HT3R, a cation channel permeable to Na^+^, K^+^, and Ca^2+^, induces rapid neuronal depolarization followed by desensitization [128]. In dentate gyrus (DG) basket cells and hippocampal interneurons, 5-HT3R-mediated currents exhibit voltage- and Ca^2+^-dependent behavior with a reversal potential near 0 mV. At hyperpolarized potentials, a negative slope conductance arises primarily due to Ca^2+^ block, suggesting a potential role as coincidence detectors for serotonergic and excitatory input [222,223]. This calcium block is further modulated by intracellular phosphates through non-phosphorylation-dependent mechanisms [224]. 5-HT3R activity is dynamically regulated by serotonergic signaling. For instance, 5-HT1AR activation reduces 5-HT3R function via phosphorylation [225], possibly shifting serotonergic modulation from excitation to inhibition. Both receptors are also subject to desensitization or downregulation with prolonged serotonin exposure [226].

In the hippocampus, 5-HT3Rs are primarily localized on dendrites of GABAergic interneurons [227,228], especially those expressing cholecystokinin (CCK) and calbindin, but not somatostatin or parvalbumin [229]. Presynaptic 5-HT3Rs on GABAergic varicosities promote GABA release via Ca^2+^ influx [201,230], with Na^+^ influx also contributing during development [22]. These receptors modulate CA3-CA1/DG signaling without markedly affecting cortical input [231] and are commonly co-expressed with CB1 [232]. Notably, they are absent from glutamatergic neurons [233]. Pharmacological blockade of 5-HT3R reduces interneuron firing and increases pyramidal neuron excitability [142]. However, 5-HT3R agonism produces complex outcomes: 2-methyl-5-HT reduces both EPSPs and IPSPs in CA1 pyramidal neurons [143], possibly via suppression of excitatory input to both interneurons and pyramidal cells [144]. Other reports show increased IPSPs upon 5-HT3R activation [136], suggesting enhanced spontaneous interneuron activity but reduced evoked GABAergic transmission [141].

Crucially, species-specific differences exist in the cellular distribution of 5-HT3Rs. While in rodents these receptors are predominantly expressed on inhibitory interneurons, in the human hippocampus they are mainly localized to pyramidal neurons [159]. This distinction carries significant implications for interpreting the physiological roles of 5-HT3Rs and evaluating their translational relevance as pharmacological targets, as differences in cellular localization may yield divergent effects on hippocampal network function across species. Notably, 5-HT3Rs are co-expressed with CB1 and α7 nicotinic receptors on CCK+ interneurons [232,234], all of which are dysregulated in schizophrenia [58,59,235]. This convergence suggests that hippocampal dysfunction in schizophrenia may, in part, stem from impaired inhibitory signaling within this distinct interneuron population. Given that 5-HT3Rs are excitatory cation channels, their modulation can rapidly influence neuronal excitability, offering therapeutic potential. For example, 5-HT3 receptor antagonists have been shown to improve working memory deficits induced by cholinergic blockade [65] and to alleviate scopolamine-induced impairments in spatial learning [153].

#### 2.2.4. 5-HT4Rs

Hippocampal pyramidal neurons co-express both 5-HT1ARs and 5-HT4Rs, which exert opposing effects on neuronal excitability. While 5-HT1ARs mediate inhibitory responses, 5-HT4Rs are responsible for the slow excitatory response to serotonin [236,237]. Additionally, co-localization of 5-HT1BRs and 5-HT4Rs has also been identified in hippocampal pyramidal cells [238]. Optogenetic stimulation of serotonergic axon terminals in the hippocampus potentiates CA3–CA1 synaptic transmission and contributes to memory formation through activation of 5-HT4R [239]. Notably, in contrast to the 5-HT1AR-dependent transient hyperpolarization induced by in vitro serotonin application, 5-HT4R-mediated excitatory effects dominate under physiological serotonergic release, highlighting the importance of spatial and temporal dynamics in neuromodulatory signaling. Activation of 5-HT4Rs also induces long-lasting EPSP–spike (E–S) potentiation in CA1 pyramidal neurons, likely through inhibition of a Ba^2+^-sensitive inwardly rectifying potassium current [240]. Similarly, endogenous serotonin elevation via MDMA application elicits E–S potentiation in the ventral hippocampus, although this effect is largely reversible [241]. Furthermore, activation of 5-HT4Rs in the CA1 region enhances spontaneous epileptiform activity in a magnesium-free medium, indicating a potential role in hyperexcitability conditions [242].

In the hippocampus, 5-HT4R activation modulates electrically evoked GABA release in a biphasic manner, without affecting basal GABAergic activity [243]. Specifically, low concentrations of agonists enhance GABA release, while high concentrations inhibit it. This effect is mediated by 5-HT4R-driven enhancement of cholinergic transmission, which subsequently activates M1/M3 muscarinic receptors at low concentrations and M2 muscarinic receptors at higher concentrations. 5-HT4Rs are involved in a wide range of physiological processes, with a predominant role in cognitive functions [244]. In the hippocampal CA1 region, they contribute to memory formation [245], counteract memory deficits induced by sleep deprivation [246], promote learning-induced dendritic spine growth [247], and support cognitive enhancement [248]. 5-HT4Rs represent a key excitatory component of hippocampal circuits, acting in opposition to the inhibitory effects mediated by 5-HT1AR activation [236,237]. Region-specific modulation of 5-HT4Rs in the hippocampus may hold particular therapeutic relevance for schizophrenia. Specifically, targeting ventral 5-HT4Rs could influence emotional regulation, while modulation of dorsal 5-HT4Rs may enhance cognitive performance [62,163,166,168,169].

#### 2.2.5. 5-HT5Rs

5-HT5Rs [79] are the least explored among serotonin receptor subtypes, and as a result, their signaling pathways and physiological roles remain largely unclear. In the hippocampus, both isoforms of the 5-HT5R have been identified: 5-HT5AR is expressed in the DG, CA3, and CA1 regions [249], while 5-HT5BR is expressed predominantly in the CA1 region [72,250]. In the DG, parvalbumin-expressing interneurons contain functionally silent 5-HT5ARs, which become active, presumably via translocation to the plasma membrane, following chronic, but not acute, SSRI treatment. This activation contributes to the effects of long-term antidepressant therapy by reducing the firing activity of PV-containing interneurons [84]. Additionally, serotonin administration reduces 5-HT5AR density in the DG while leptin treatment decreases receptor density in both the CA1 region and DG [251]. Although these changes were not statistically significant, similar trends were observed across all hippocampal subregions, suggesting that 5-HT5ARs may influence hippocampal neurogenesis, particularly since leptin has been shown to promote neurogenic processes in the DG [252]. Although the physiological role of hippocampal 5-HT5Rs remains the least characterized among serotonin receptor subtypes, evidence suggests their involvement in several central functions, including motor activity, memory, sleep regulation, mood, and emotional behavior [170]. Moreover, 5-HT5R modulators have demonstrated procognitive and antidepressant-like effects in preclinical models [253], indicating potential therapeutic relevance for memory deficits in neuropsychiatric and neurodevelopmental conditions such as dementia [173], schizophrenia [174,175], and autism spectrum disorders [9].

#### 2.2.6. 5-HT6Rs

In the hippocampus, 5-HT6R mRNA is found in both pyramidal neurons and GABAergic interneurons but is absent from neuromodulatory neurons [254]. The 5-HT6R is predominantly expressed on pyramidal projection neurons [255]. Additionally, about half of the 5-HT3R-positive interneurons co-express the 5-HT6R, whereas expression in parvalbumin-containing and somatostatin-containing interneurons is negligible. In contrast, most cholecystokinin-expressing interneurons express the 5-HT6R. The highest density of 5-HT6Rs is observed in the molecular layer of the DG and in the CA1 stratum oriens and stratum radiatum, where the receptors exhibit a dendritic localization [256]. Recent evidence suggests that 5-HT6R activation shifts the hippocampal excitation–inhibition balance toward inhibition. Specifically, in the dorsal hippocampus, activation of 5-HT6R significantly increases GABA levels and reduces stimulus-evoked glutamate release, though it does not alter basal glutamate levels [257]. Moreover, presynaptic 5-HT6Rs located on axon terminals inhibit glutamate release by suppressing vesicular exocytosis [258]. Conversely, in the CA1 region, blockade of 5-HT6Rs increases basal excitatory transmission and NMDA receptor activation, without affecting synaptic plasticity [259]. Interestingly, these effects were prevented by GABA_A_ receptor antagonists in male but not female mice, suggesting a sex-dependent modulation of hippocampal excitability by 5-HT6Rs.

As will be discussed in Section 4.1, altered 5-HT6R expression has been reported in both animal models and postmortem brain tissue from patients with schizophrenia. Pharmacologically, 5-HT6R antagonists enhance working memory and cognitive flexibility, supporting their potential utility in addressing cognitive deficits in schizophrenia [176,260]. In addition to cognition, hippocampal 5-HT6Rs have been implicated in emotional regulation and depression, suggesting that their modulation may also ameliorate emotional dysregulation or depressive comorbidity commonly observed in schizophrenia [177,178,179,180,181].

#### 2.2.7. 5-HT7Rs

In the hippocampus, 5-HT7Rs are highly expressed in the CA3 region, with lower expression levels in CA1, CA2 [261], and the DG [262]. Activation of 5-HT7Rs increases population spikes and enhances excitability in the CA1 region [263], while blockade of these receptors abolishes their excitatory effects on hippocampal network activity [264]. Serotonergic input selectively suppresses perforant path input, but not Schaffer collateral input to the CA1 region [265]. This suppression appears to be postsynaptic and is partially mediated by 5-HT2ARs and 5-HT7Rs [265]. In the CA3 area, 5-HT7R activation increases bursting activity, likely via a reduction in the afterhyperpolarization through inhibition of a calcium-activated potassium conductance [266]. In the ventral CA3 region, activation of 5-HT7Rs enhances action potential frequency by stimulating I_h_ current and has been shown to facilitate fear memory retrieval [267]. Regarding the GABAergic system, 5-HT7R activation in the CA1 region enhances inhibitory transmission through a dual mechanism: increasing glutamatergic drive onto interneurons and directly facilitating GABA release from axon terminals [268]. In pathological conditions, blockade of 5-HT7R reduces epileptic activity [269], and in a pilocarpine-induced temporal lobe epilepsy model, a reduction in 5-HT7R density has been observed in the hippocampus, particularly in the DG [270].

**Figure 2:**
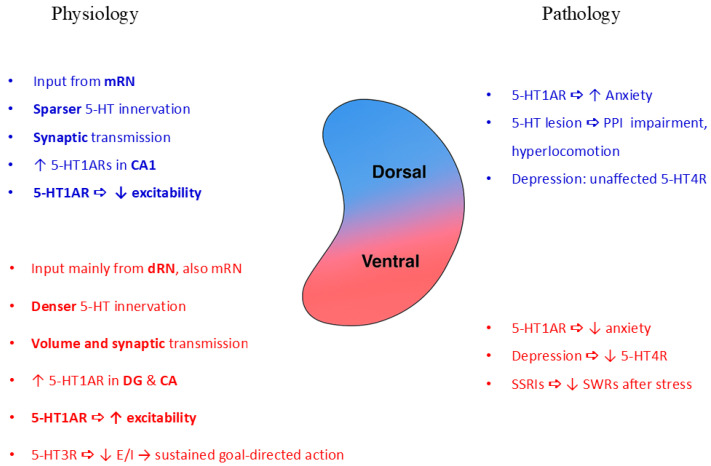
Roles of the serotonergic system in regulating the dorsal and ventral hippocampus in physiological and pathophysiological states. Arrows indicate the direction of change. ↑ represents an increase or enhancement, and ↓ represents a decrease or reduction in the respective parameter.

In the dorsal hippocampus, functional interplay between 5-HT1ARs, 5-HT7Rs, and GABARs has been implicated in the modulation of learning processes [188]. Additionally, 5-HT7Rs contribute to antidepressant effects by facilitating emotional learning within the hippocampus [189]. In models of neuropsychiatric disorders, 5-HT7R activation exerts anxiolytic effects and elevates 5-HT, noradrenaline, and dopamine levels specifically in the ventral hippocampus [194], suggesting regionally distinct effects. Furthermore, 5-HT7R stimulation has demonstrated therapeutic potential in reversing molecular and behavioral phenotypes in neurodevelopmental disorders such as Rett syndrome [195]. Taken together, these findings suggest that dorsoventral modulation of 5-HT7Rs may have symptom-specific therapeutic implications in schizophrenia: dorsal 5-HT7R activation could enhance cognitive functions, while ventral 5-HT7R stimulation may alleviate emotional dysregulation. Building on this, pharmacological targeting of hippocampal 5-HT7Rs could help restore neurotransmitter imbalances across the longitudinal axis in patients with schizophrenia and mitigate both cognitive and affective symptoms [271]. Figure 2 summarizes the dorsoventral differences in serotonergic modulation of the hippocampus.

Intending to complement the summarized functional overview shown in Figure 2, Table 3 summarizes the regional distribution, major cellular expression, effects on circuitry, and roles in schizophrenia symptoms, and available or potential therapeutic implications, of 5-HTR subtypes comparatively in the dorsal and ventral hippocampus.

Taken together, 5-HT1ARs, 5-HT2ARs, and 5-HT3Rs exhibit distinct regional localizations and circuit actions along the hippocampal dorsoventral axis. While dorsal 5-HT1AR activity predominantly exerts inhibitory control linked to cognition, ventral 5-HT2AR and 5-HT3R signaling promote excitation associated with emotional and psychotic phenomena. However, comparative analysis across animal models and patient studies reveals species- and context-dependent differences in cellular localization and symptom relevance. These findings underline the need for region- and receptor-specific approaches in both mechanistic studies and therapeutic development, particularly in light of unresolved controversies regarding receptor expression patterns and function in schizophrenia.

## 3. Serotonergic Regulation of Hippocampal Network Dynamics

Serotonin modulates neuronal circuit activity through its cellular and synaptic actions, shaping network excitation, synchronization, synaptic plasticity, and oscillatory dynamics. This section will describe the actions of serotonin and its receptors on synaptic plasticity and network dynamics in the hippocampus.

### 3.1. Synaptic Plasticity

Long-term synaptic plasticity is considered a fundamental mechanism underlying learning and memory, typically manifested as long-term potentiation (LTP) and long-term depression (LTD). Although long-term synaptic plasticity is closely linked to NMDA receptor signaling, there are various forms of long-term potentiation associated with GABAergic, cholinergic, and serotonergic receptors [272]. The role of serotonin in long-term synaptic plasticity remains incompletely understood. On one hand, endogenous serotonin has been shown to facilitate hippocampal LTP at CA3–CA1 synapses [273], activation of the raphe nuclei enhances LTP expression in the dentate gyrus [274], and depletion of 5-HT reduces LTP in the dentate gyrus [275]. On the other hand, exogenously applied serotonin has been shown to prevent the induction of LTP by primed burst stimulation, presumably through activation of 5-HT1AR and 5-HT3R [276]. Additionally, serotonin dose-dependently reduces LTP at CA1 synapses [277] and at commissural/associational CA3 synapses [278]. These seemingly contradictory findings suggest that different concentrations of serotonin may either promote or inhibit LTP, potentially through synergistic or antagonistic actions on various 5-HT receptor subtypes. In the CA1 hippocampal region, the SSRI fluvoxamine suppresses LTP via 5-HT1AR [7]. Interestingly, following contextual fear conditioning, elevated extracellular levels of 5-HT and suppressed LTP in the CA1 region were observed only in the hippocampus of male, but not female, rats, a finding likely related to 5-HT1AR [279]. These results suggest a sex-specific serotonergic modulation of hippocampal-dependent emotional responses. However, at perforant path–granule cell synapses in the DG, 5-HT1ARs appear to facilitate LTP, as chronic administration of YL-0919, a combined selective 5-HT reuptake inhibitor and partial 5-HT1A agonist, has been shown to enhance LTP [280].

Activation of 5-HT2Rs following MDMA application in hippocampal slices enhances LTP at CA3–CA1 synapses [281] and reverses the impairment of hippocampal LTP induced by acute stress exposure [282]. Deletion of 5-HT3AR impairs the NMDA-dependent LTD, but not the mGluR-dependent LTD, in the CA1 region, and blockade of 5-HT3R reduces the internalization of AMPA receptors [283]. Blockade of 5-HT3R facilitates the induction of LTP in the hippocampus of freely moving rats [284], while activation of 5-HT3R in BDNF^Met/Met^ mice reverses the impaired hippocampal LTD [285,286]. Furthermore, activation of 5-HT3R inhibits the induction of LTP at the mossy fiber–CA3 synapses by enhancing GABAergic transmission and reducing cholinergic signaling [285].

5-HT4R differentially modulates long-term synaptic plasticity across distinct hippocampal subregions. More specifically, activation of 5-HT4R in the CA1 region prevents LTP depotentiation and suppression of LTD, without affecting LTP in vivo [287]. Additionally, activation of 5-HT4R facilitates the induction of LTP in response to previously subthreshold stimulation, whereas blockade of this receptor leads to LTD. In contrast, in vitro studies have shown that the activation of 5-HT4R decreases theta-burst stimulation-induced LTP [288]. Activation of 5-HT4R prevents the induction of both LTP and LTD at mossy fiber–CA3 synapses, blocks LTD, and attenuates established LTP at perforant path–dentate gyrus (PP–DG) synapses [289]. Overall, 5-HT4R appears to promote LTP at the expense of LTD in the dentate gyrus and CA1 regions, while preventing both forms of long-term synaptic plasticity in the CA3 region [244]. Activation of 5-HT6R in the dorsal CA1 augments baseline GABAergic neurotransmission and attenuates LTP [290], while blockade of this receptor prolongs LTD at PP-DG synapses [289] but enhances LTP in a pilocarpine-induced model of epilepsy [185]. Finally, activation of 5-HT7R reduces the mGluR-mediated LTD at the CA3-CA1 synapses in both wild-type and *Fmr1* knock-out mice [291] and restores the impaired LTP at PP-DG synapses in a rat model of Alzheimer’s disease [292].

Briefly, in the CA1 hippocampal region, 5-HT2R, 5-HT4R, 5-HT6R, and 5-HT7R increase the magnitude of LTP, while 5-HT1AR and 5-HT3R have the opposite effect. In the CA3 region, 5-HT3R reduces LTP, while 5-HT4R prevents long-term plasticity. In the DG, 5-HT1AR, 5-HT4R, 5-HT6R, and 5-HT7R increase LTP. Therefore, serotonin can regulate long-term synaptic plasticity in the hippocampus in a complex manner, depending on the spatial and temporal pattern of receptor activation. This regulation is particularly important, as dysconnectivity, whether anatomical (e.g., aberrant wiring during brain development) [293] or functional (e.g., impairments in synaptic plasticity) [294], is thought to contribute to the pathophysiology of schizophrenia [295]. More specifically, according to the functional disconnection hypothesis [296], the core pathological mechanism in schizophrenia involves aberrant NMDA receptor-mediated synaptic plasticity, resulting from its dysregulated modulation by neuromodulatory transmitters such as 5-HT, ACh, and DA. Dysfunctional synaptic plasticity, whether enhanced or diminished, is a characteristic feature of the hippocampus in relation to schizophrenia-like deficits [297]. For instance, studies using the NMDA receptor antagonist MK-801 to model schizophrenia have demonstrated impaired PP-DG LTP [298,299], impaired SC-CA1 LTP [300], and enhanced CA1–subiculum LTP [301]. Accordingly, the modulatory effects of serotonergic receptors, such as 5-HT2AR and 5-HT7R, on glutamate levels and consequently on NMDA receptor activation could promote a state of metaplasticity with potential beneficial effects on neuropsychiatric symptoms [302]. Furthermore, genetic rodent models of the disorder show either enhanced [303] or reduced [304] LTP at CA3–CA1 synapses.

### 3.2. Hippocampal Rhythms

Synchronization of neural activity is essential for proper circuit function, and hippocampal network oscillations have been proposed as biomarkers of neuropsychological disorders [305]. The hippocampus exhibits three major types of network patterns—theta rhythm, gamma rhythm, and sharp wave–ripple complexes—each serving distinct functional roles in brain processes and behavior [306]. Hippocampal oscillations result from precisely coordinated neural activity, and serotonin shapes the properties of these rhythms by activating receptors on both GABAergic interneurons and pyramidal neurons.

#### 3.2.1. Theta Rhythm

Theta rhythm is a low-frequency (3–8 Hz) oscillatory activity that occurs during active exploration and rapid eye movement sleep [306,307]. It plays important roles in episodic, spatial, and working memory, adaptive learning, relational binding, social cognition, and flexible decision making [308]. In the hippocampus, serotonin decreases theta rhythm activity. Stimulation of mRNu desynchronizes the dorsal hippocampal theta activity [309], and median raphe lesions enhance it [310]. Interestingly, specific median raphe serotonergic fast-firing neurons are phase-locked with the hippocampal theta activity, while slow-firing serotonergic neurons are presumably important for maintaining a basal serotonergic input to the hippocampus [311]. Furthermore, serotonin depletion facilitates place learning and is associated with earlier and higher expression of theta activity in the CA1 area of the hippocampus after the learning period [312]. SSRI administration reduces theta oscillations in both the dorsal and ventral hippocampus in vivo [313]. Pharmacological activation of 5-HT1AR reduces hippocampal theta rhythm [8], but see also [314]. Blockade of 5-HT2AR [8] and 5-HT2CR suppresses theta oscillations [315], while blockade of 5-HT3R enhances theta activity in freely moving rats [284] and increases the donepezil-induced augmentation of theta rhythm in the dorsal hippocampus [316]. Activation of 5-HT4R enhances the hippocampal theta oscillations [317]. In freely moving rats, activation of 5-HT6R reduces the frequency, but not the power, of theta activity [318]. In contrast, blockade of 5-HT6R does not affect theta activity, suggesting that these receptors do not exert a tonic influence on theta oscillations [318]. Furthermore, blockade of 5-HT6R potentiates the donepezil-induced enhancement of theta activity [319].

#### 3.2.2. Gamma Rhythm

Gamma rhythm is a high-frequency oscillatory activity, typically divided into slow gamma (30–60 Hz) and fast gamma (60–100 Hz), that has been implicated in a range of cognitive functions, including working memory, spatial navigation, selective attention, sensory gating, and perceptual integration [320,321,322]. Serotonergic modulation exerts complex and region-specific effects on gamma oscillations within the hippocampus. Administration of SSRIs reduces gamma activity in both the dorsal and ventral hippocampus [313]. Similarly, increased serotonin levels suppress kainate-induced gamma power while enhancing stimulus-induced gamma oscillations [323]. Activation of 5-HT1ARs has been shown to reduce both multi-unit activity and gamma oscillations in the dorsal hippocampus [8]. This suppression is also observed in the CA3 [324] and CA1 [314] regions. However, 5-HT1AR activation can enhance gamma coupling between the ventral CA1 and the prefrontal cortex [314], indicating pathway-specific effects. In the ventral hippocampus, serotonin decreases the power, but not the frequency, of carbachol-induced gamma oscillations (20–40 Hz) via 5-HT1ARs, while 5-HT2R activation produces the opposite effect [325]. Consistently, 5-HT2AR blockade reduces gamma activity in the dorsal hippocampus [8].

The role of 5-HT3Rs located on CCK-containing GABAergic interneurons is particularly notable. Their activation disrupts gamma synchronization by enhancing spike-frequency adaptation, which in turn increases firing in PV-containing interneurons, ultimately disturbing network oscillations [326]. Blocking 5-HT3Rs enhances the gamma- and theta-augmenting effects of donepezil, a cholinesterase inhibitor, although 5-HT3R blockade alone has no such effect [316]. Similarly, BDNF-facilitated gamma enhancement is thought to result from reduced 5-HT3R signaling [327]. Blockade of 5-HT6Rs also potentiates donepezil-induced gamma oscillations [319]. However, in apparent contrast, simultaneous blockade of 5-HT3Rs and 5-HT6Rs reduces PCP-induced augmentation of gamma and high-frequency oscillations in the dorsal hippocampus [328]. This discrepancy suggests that the physiological context, such as increased acetylcholine levels or NMDA receptor antagonism, plays a critical role in shaping how serotonergic receptors influence hippocampal gamma activity. In alternative circuit states, blockade of 5-HT3Rs and 5-HT6Rs can either enhance or suppress gamma oscillations, possibly due to their expression on GABAergic interneurons [229,254]. Co-inhibition may dampen interneuron activation to a degree that disrupts overall network synchronization. Notably, hippocampal gamma rhythms are consistently altered in patients with schizophrenia and animal models of schizophrenia [308]. While elevated gamma activity is more commonly reported, certain models also exhibit reduced gamma oscillations. This variability supports the notion that disruption of serotonergic modulation in the hippocampus may contribute to aberrant gamma rhythms, potentially underlying cognitive and perceptual disturbances observed in schizophrenia.

#### 3.2.3. Sharp Waves and Ripples (SWRs)

Sharp waves–ripples (SWRs) are intrinsic hippocampal activity patterns involved in a range of cognitive and behavioral functions, including memory consolidation, stress and anxiety regulation, social memory, decision-making, and mind wandering [329,330,331,332,333,334,335]. An SWR event comprises a slow potential shift, known as the sharp wave, overlaid by a high-frequency oscillation called the ripple (~150 Hz). The firing activity of neuronal cell assemblies during SWRs is highly organized, representing spatiotemporally structured reactivations of pyramidal cells—sequences that were initially formed during previous experiences [329,336,337]. SWRs also exhibit distinct characteristics along the dorsoventral axis of the hippocampus, suggesting functional specialization between the dorsal and ventral regions [338,339]. The generation of normal SWRs depends on a finely tuned E/I balance [329,340]. Disruption of this balance, which is thought to underlie several neurodevelopmental disorders such as schizophrenia, is expected to impair both the physiological generation of SWRs and SWR-associated information processing [212,341,342,343,344,345].

Under in vivo conditions, many median raphe neurons are inactive during ripple oscillation, while ripples are enhanced and suppressed by optogenetic inactivation and stimulation of median raphe neurons, respectively [346]. Furthermore, the blockade of hippocampal 5-HT1ARs in vivo reduces the number of ripple events, while blockade of 5-HT3Rs has the opposite effect [347]. Application of serotonin in dorsal hippocampal slices masks SWRs induced by tetanic stimulation in a dose-dependent manner [348]. This effect seems to be mediated by 5-HT1ARs and 5-HT2A/CRs but not 5-HT3Rs or 5-HT4Rs. Additionally, endogenous serotonin suppresses SWRs in the DG [349]. Finally, stress exposure increases SWRs in the ventral, but not dorsal, hippocampus, and administration of SSRIs selectively reduces the SWRs in the ventral part of the hippocampus, suggesting a mechanism for the antidepressant effects of SSRIs [313]. In general, although not extensively studied, serotonin seems to decrease the SWR activity. More studies are necessary to reveal the roles of specific serotonergic receptors on SWRs with an emphasis on possible dorsoventral differences of the hippocampus. Unpublished data from our laboratory suggest that serotonin differentially modulates SWRs in the dorsal and ventral hippocampus.

In patients with schizophrenia, SWRs occur more frequently, but in a disorganized spatiotemporal pattern, and have been associated with positive symptoms [350]. Similarly, in maternal immune activation (MIA) models of schizophrenia, animals display more frequent SWRs in the dorsal hippocampus, along with increased SPW amplitude and ripple strength [351,352]. Genetic models also show elevated ripple power [353,354], although ketamine administration has been reported to reduce SWR frequency in the dorsal hippocampus [355]. The effects of 5-HTRs on hippocampal network dynamics are summarized in Table 4.

### 3.3. Developmental Aspects of Serotonergic Regulation

During development, serotonin plays a dual role, functioning both as a classical neurotransmitter and as a regulator of neurodevelopmental processes, particularly within the hippocampus. In this context, several psychiatric disorders, including schizophrenia and autism spectrum disorders, have been linked to disruptions in neurodevelopment during both fetal and early postnatal life. Serotonergic fibers begin innervating the hippocampus by embryonic day (E) 19, with tryptophan hydroxylase (TPH) activity and serotonin levels peaking during the early postnatal period [356,357,358]. Monoamine oxidases (MAO-A/B) and transient neuronal expression of the serotonin transporter (SERT) further modulate local serotonin dynamics [359,360]. The timely expression of serotonin receptors (e.g., 5-HT1AR, 5-HT4R, 5-HT6R) coordinates key processes such as dendritic growth, neuronal migration, and synaptic maturation [23,361,362]. These findings underscore serotonin’s essential role as a neurodevelopmental signal, with implications for understanding developmental disorders linked to serotonergic dysfunction. For instance, exposure to stress during early developmental stages, a known risk factor for many psychiatric disorders, alters the serotonergic system in the hippocampus. For example, early-life stress during the second postnatal week disrupts the function of 5-HT1ARs in relation to long-term synaptic plasticity in adult rats, whereas stress during the third postnatal week has no such effect [363]. In another study, stress exposure during the third postnatal week reduced 5-HT1AR levels in the dorsal hippocampus of post-adolescent rats [364]. Interestingly, while early-life stress reduces 5-HT1AR expression in adult rats, it was found to elevate 5-HT1AR mRNA levels in the CA1 region of developing rats [365]. Moreover, either juvenile or adult stress alone decreases 5-HT1ARs and 5-HT3R-mediated modulation of inhibition in ventral DG granule cells without affecting emotional behavior [366]. Surprisingly, when both juvenile and adult stress are experienced, these serotonergic changes are reversed, but this combination leads to robust anxiety-like behavior. As suggested by the authors, the stress-induced reduction in serotonergic inhibition in granule cells of the ventral DG may serve as a resilience mechanism, which is disrupted following a second stressful event. Another interesting aspect of the serotonergic system in respect to the stress response is its divergent adaptation after acute and chronic stress, including changes in serotonin metabolism and turnover [367].

## 4. The Hippocampal Serotonergic System in Schizophrenia

From the above discussion, three main conclusions can be drawn. First, the serotonergic system comprises a wide variety of receptors that modulate the activity of pyramidal cells, interneurons, and other neurotransmitter systems. This complexity is essential for the spatially and temporally precise responses to environmental cues and internal mental states. Second, considering the functional segregation along the dorsoventral axis of the hippocampus, the serotonergic system may contribute more prominently to the modulation of emotional processes (e.g., stress regulation, fear memory, social memory) via the ventral hippocampus, while it may influence cognitive processes primarily through the dorsal hippocampus. Third, the effects of serotonin on the hippocampus can be conceptualized within the framework of network excitability. By regulating the activity of excitatory and inhibitory neurons, modulating synaptic strengths, and synchronizing neuronal populations, the serotonergic system helps maintain the balance between excitation and inhibition. Conversely, disruption of the serotonergic system can destabilize the proper function of the hippocampus, leading to the emergence of neuropsychiatric symptoms. In the following sections, we review schizophrenia-related alterations of the serotonergic system in the hippocampus, attempting to integrate the basic physiological actions of serotonin with the pathophysiology of hippocampal circuits, thus providing a coherent framework that bridges basic mechanisms with the emergence of schizophrenia symptoms.

The role of the hippocampus in the pathophysiology and symptomatology of schizophrenia is well-established [12]. One of the most consistent neuroanatomical findings is a reduction in hippocampal volume, detectable even during the first psychotic episode [32,33]. Hippocampal volume loss has been proposed as a potential biomarker for cognitive outcomes in various neuropsychiatric disorders [57]. Accordingly, cannabis use, one of the risk factors for psychosis, has been associated with structural alterations of the hippocampus [58,59]. Given that schizophrenia typically manifests in late adolescence or early adulthood, the disorder is widely believed to have a significant neurodevelopmental component [368]. As with many neurodevelopmental disorders, alterations in the E/I balance are considered a central pathophysiological mechanism [209,210,211]. Disruption of the E/I balance, particularly in the hippocampus, may play a key role in the onset and progression of schizophrenia [212], contributing to both positive symptoms and cognitive deficits [213]. One influential hypothesis posits that NMDA receptor hypofunction on GABAergic interneurons in the hippocampus leads to disinhibition and elevated tonic activity of excitatory pyramidal neurons [60]. Supporting this, transplantation of GABAergic interneurons into the ventral hippocampus of schizophrenia animal models has been shown to reduce hippocampal hyperactivity and improve both cognitive and negative symptoms [36]. Notably, schizophrenia-related ventral hippocampal overactivity may drive increased mesolimbic dopaminergic tone, contributing to aberrant dopamine signaling [60]. Furthermore, it has been hypothesized that schizophrenia may begin with excitotoxic damage to the CA1 hippocampal region, eventually leading to psychosis and hippocampal atrophy [369]. This excitotoxicity, particularly in early disease stages, could be driven by excessive serotonergic input from the dRN following stress exposure, resulting in elevated hippocampal glutamate levels [370]. While early excitotoxicity may result from excessive serotonergic input, postmortem studies of schizophrenic patients show decreased 5-HT levels [18] and reduced paroxetine binding affinity on the SERT [17,19], suggesting a subsequent decline in serotonergic tone. Accordingly, while a hyperserotonergic state has been associated with the onset of the disorder, reduced serotonin levels in later stages may contribute to the expression of positive symptoms.

The schizophrenia-related alterations in hippocampal serotonin function are often linked to environmental risk factors, and the consequent hippocampus dysfunction may underlie core symptoms of the disorder. Also, proinflammatory mediators promote tryptophan degradation and serotonin catabolism [371], contributing to reduced 5-HT availability. In hyperdopaminergic rat models, elevated hippocampal MAO-B levels may also lead to increased serotonin breakdown [372]. This is particularly relevant given that limbic hyperdopaminergia is a hallmark of schizophrenia. In addition, in STOP protein-deficient mice, serotonin levels are reduced in the hippocampus, and raphe nuclei are dysregulated [373]. Social isolation, another environmental risk factor, decreases serotonin efflux in the hippocampus [374]. Reduced SERT density has also been observed in STOP-deficient mice, which may reflect either loss of serotonergic innervation or compensatory downregulation in response to low 5-HT levels. Conversely, prenatal stress increases SERT and c-FOS expression in the hippocampus [375], pointing to complex, possibly context-dependent regulation. Additional disruptions are observed in TPH2, the key enzyme in serotonin synthesis. TPH2 levels have been reported as both elevated and decreased in schizophrenia models. For instance, maternal high-fat diets, which induce inflammatory states, increase TPH2 expression in the adolescent hippocampus without altering serotonin levels [376], while STOP-deficient mice exhibit decreased TPH2 activity [373]. Notably, serotonin depletion in the hippocampus, unlike in the cortex, does not trigger compensatory TPH2 upregulation [377], possibly leading to a hypersensitive serotonergic state. This is supported by findings in dysbindin-deficient mice, which show exaggerated responses to serotonin in hippocampal slices [378]. Altogether, these findings suggest that reduced SERT expression may indicate either aberrant serotonergic input or homeostatic adaptation, both contributing to hippocampal dysfunction. Importantly, serotonergic disturbances impair not only synaptic function but also developmental processes, particularly when coinciding with critical neuroplastic periods such as childhood or adolescence. In such vulnerable windows, environmental stressors may provoke excessive raphe nucleus activity, elevating 5-HT levels, which, in a hypersensitized hippocampus, could lead to excitotoxic injury. Considering the dorsoventral axis of the hippocampus, lesions in serotonergic projections from the dRN to the ventral hippocampus impair prepulse inhibition (PPI), while lesions from the mRN to the dorsal hippocampus result in locomotor hyperactivity and PPI deficits [379], both behaviors relevant to schizophrenia. These findings underscore the region-specific involvement of the hippocampus in the disorder [37,38].

The cholinergic system in the hippocampus has also been implicated in schizophrenia [380,381] and is now recognized as a pharmacological target [382,383]. One proposed model suggests that a hyperserotonergic hippocampal state, possibly caused by reduced SERT function, enhances acetylcholine release via serotonergic heteroreceptors. This, in turn, could downregulate M1/M4 muscarinic receptors, contributing to schizophrenia-like cognitive impairments [384]. Relevant serotonergic receptors involved include 5-HT1AR, 5-HT2AR, 5-HT3R, and 5-HT4R, all of which are known to facilitate acetylcholine release in the hippocampus. Additionally, α7 nicotinic receptors, which are implicated in interneuronal deficits in schizophrenia [235], are co-expressed in CCK-containing interneurons that also express 5-HT3Rs [234]. Cholinergic α7 receptors modulate PPI, control long-term synaptic plasticity along the dorsoventral hippocampal axis [385], and regulate hippocampal oscillatory rhythms [386]. Agonism of M1 and M2 muscarinic receptors has also shown potential to ameliorate schizophrenia symptoms, particularly cognitive deficits [387]. In conclusion, converging evidence from molecular, anatomical, and functional studies supports the notion that disrupted serotonergic regulation, alongside its interactions with the cholinergic system, within the hippocampus plays a fundamental role in the etiology and symptomatology of schizophrenia. This dysfunction may underlie a wide range of clinical features, from cognitive impairments and disorganized behavior to stress sensitivity and altered neural circuitry, offering multiple avenues for targeted therapeutic intervention.

### 4.1. 5-HTRs in Schizophrenia

One of the most prominent serotonergic alterations observed in schizophrenia is the dysregulation of 5-HTR expression. Multiple studies in both patients and animal models have reported changes in 5-HTR subtypes, and pharmacological manipulation of these receptors via activation or blockade can replicate or alleviate various schizophrenia-related symptoms. Among these, 5-HT1ARs and 5-HT2ARs are the most extensively studied, likely because they are primary targets of atypical antipsychotic medications. For instance, isolation rearing, a known environmental risk factor for schizophrenia that induces hyperactivity, PPI deficits, and hippocampal atrophy, has been shown to increase hippocampal 5-HT1AR expression [374]. Similarly, prenatal infection, another risk factor, leads to elevated 5-HT1AR levels specifically in the CA1 region of the hippocampus. Postmortem studies in patients with schizophrenia have demonstrated significantly elevated 5-HT1AR density in the DG, with trends toward increased expression in CA1 and CA3 as well [388]. Another study found no statistically significant differences in hippocampal 5-HT1AR levels, though a general tendency for elevation was again noted [384].

Pharmacological models offer additional support for these findings. Administration of MK-801, an NMDA receptor antagonist used to simulate schizophrenia-like symptoms, increased 5-HT1AR binding sites in the ventral CA1, without affecting mRNA levels [10]. Notably, blockade of 5-HT1ARs mitigates MK-801-induced hyperactivity, improves sensorimotor gating, and restores working memory performance [389]. Systemic administration of a 5-HT1AR antagonist that effectively abolishes 5-HT1AR functionality and elevates synaptic serotonin has been shown to reduce locomotor activity and decrease D2/3 receptor binding in the ventral but not dorsal hippocampus, presumably via enhanced dopamine release [390]. Interestingly, laterality may influence serotonergic modulation of hippocampal function. For example, 5-HT1AR blockade produced a stronger reduction in locomotor activity when applied to the right hippocampus, suggesting a left–right asymmetry in receptor function [391]. In contrast, pharmacological activation of 5-HT1ARs has been shown to disrupt PPI, likely via activation of postsynaptic receptors, rather than presynaptic autoreceptors in the raphe nuclei [392]. Activation of 5-HT1ARs and 5-HT1BRs in the dorsal CA1 region reduces object exploration and habituation, with 5-HT1BR activation additionally inducing phobic responses to novel stimuli and reducing locomotor activity [66]. Supporting these behavioral effects, elevated 5-HT1BR mRNA levels have been reported in the hippocampus of schizophrenia patients [393]. These findings collectively suggest that serotonergic modulation in the hippocampus, particularly via 5-HT1ARs, affects emotional responses, environmental adaptation, and attention processing. Decreased habituation, as seen in these models, may relate to the hyper-attentiveness commonly reported in schizophrenia. Thus, upregulation of hippocampal 5-HT1ARs appears to be a consistent finding across schizophrenia studies, and blockade of these receptors tends to ameliorate associated symptoms, whereas receptor agonists exacerbate them. Of note, while most evidence regarding 5-HT1AR alterations in schizophrenia derives from rodent models, extrapolation to humans should be performed cautiously.

Regarding 5-HT2ARs, postmortem studies in patients with schizophrenia have yielded mixed results, showing both decreased [384,393] and increased [388] receptor expression in the hippocampus. These conflicting postmortem findings may reflect methodological differences, small and heterogeneous samples, or variability in medication status of the patients studied. Functionally, 5-HT2AR blockade in the ventral hippocampus has been shown to reduce PCP-induced neuronal activation, likely by dampening the excessive glutamatergic activity associated with NMDA receptor blockade [394]. Interestingly, partial agonism of 5-HT2ARs in the same region reverses PPI deficits caused by dopamine D1/D2 receptor stimulation, suggesting a modulatory role in dopamine–serotonin interactions [395]. However, under baseline conditions, 5-HT2A activation in the ventral hippocampus does not impair PPI, unlike its effects when applied to other brain regions. Similarly, blockade of 5-HT2ARs has no observable effect on PPI under normal conditions in any of the studied regions [396]. Furthermore, systemic administration of either a 5-HT2AR agonist or antagonist increases D2/3 receptor binding in the ventral hippocampus, presumably due to reduced dopamine availability, and concurrently reduces locomotor activity [397]. These findings underscore the complex role of 5-HT2ARs in schizophrenia. In the ventral hippocampus, both partial agonism and antagonism can potentially ameliorate different symptom domains, such as impaired sensorimotor gating and hyperactivity, respectively. Also, it is worth noting that while psychedelics, acting largely via 5-HT2ARs, have historically been associated with psychosis-like effects, emerging evidence suggests that low-dose psychedelics may exert beneficial effects on negative and cognitive symptoms in schizophrenia [398], although this area remains controversial and requires further investigation. The widespread distribution of 5-HT2ARs on both pyramidal neurons and GABAergic interneurons may contribute to this complex involvement of 5-HT2ARs in schizophrenia, as the dose and pharmacodynamics of 5-HT2AR-targeting drugs can elicit divergent physiological effects. Additionally, the physiological context, including the state of other neurotransmitter systems and the underlying neural substrate, modulates receptor function and further complicates interpretation.

Although less extensively studied, other serotonin receptor subtypes have also been implicated in schizophrenia. 5-HT3R antagonists, such as ondansetron, improve auditory gating, possibly via enhancement of cholinergic transmission in the hippocampus [399]. Tropisetron, another 5-HT3R antagonist, exhibits antidiabetic effects [400], which may be relevant for schizophrenia management given the high prevalence of metabolic syndrome associated with atypical antipsychotics. Notably, 5-HT3R antagonists have been proposed as promising treatments across psychiatric conditions, including schizophrenia, due to their favorable side effect profiles, potential to improve negative symptoms, and procognitive effects [401]. Downregulation of hippocampal 5-HT4R expression has been associated with anhedonia in animal models of depression [11]. Although anhedonia is a core feature of depression, it also characterizes the blunted emotional response observed in schizophrenia, suggesting a potential role for 5-HT4Rs in both disorders. 5-HT5Rs, particularly the 5-HT5AR subtype, though not specifically studied in the hippocampus, have been linked to schizophrenia. A genetic association has been reported between 5-HT5AR polymorphisms and late-onset schizophrenia [402]. Additionally, blockade of 5-HT5ARs reverses cognitive deficits and social withdrawal in ketamine-based models of schizophrenia [175]. Importantly, 5-HT5AR-targeting compounds are thought to offer antipsychotic effects with fewer adverse effects, such as sedation, elevated prolactin, or catalepsy [253].

Postmortem studies of patients with schizophrenia treated with typical antipsychotics have reported reduced hippocampal 5-HT6R mRNA levels [403]. In the hippocampus, 5-HT6Rs are located on GABAergic interneurons, and their activation is thought to reduce glutamatergic transmission, whereas blockade of 5-HT6Rs disinhibits pyramidal neurons and increases excitatory output [404]. In a double-hit neurodevelopmental model combining PCP administration with social isolation, the ability of 5-HT6R antagonism to enhance glutamatergic transmission in the dorsal hippocampus was diminished, likely due to reduced function of calbindin-positive interneurons that co-express 5-HT6 receptors [405]. Given that glutamate hypofunction in the hippocampus is linked to declarative memory deficits in schizophrenia [406], reduced 5-HT6R function may represent a compensatory mechanism aimed at elevating hippocampal glutamate levels. However, NMDA receptor antagonism does not affect 5-HT6R mRNA levels in the hippocampus [407].

In a post-weaning isolation model, 5-HT6R blockade reversed novel object discrimination deficits, possibly by increasing hippocampal glutamate and enhancing dentate gyrus neurogenesis [408]. Further, 5-HT6R blockade improves working memory performance, an effect linked to enhanced cholinergic transmission [409]. As previously discussed, blockade of 5-HT6Rs facilitates hippocampal cholinergic signaling, although it does not impact PPI [410]. Thus, 5-HT6R antagonism appears to reverse cognitive deficits in schizophrenia by enhancing both glutamatergic and cholinergic neurotransmission in the hippocampus. Similarly, in PACAP-deficient mice, a model relevant to psychiatric disorders, 5-HT7R blockade improves both psychomotor and cognitive deficits and additionally inhibits neurite outgrowth in the hippocampus [411]. Table 5 summarizes key findings from animal models and postmortem human studies that highlight alterations in hippocampal serotonergic markers associated with schizophrenia-related risk factors, pathophysiological mechanisms, and clinical phenotypes.

In parallel, astrocyte dysfunction has been implicated in schizophrenia, potentially contributing to glutamatergic dysregulation, white matter abnormalities, and impaired synaptic plasticity [412]. Furthermore, genetic risk factors for schizophrenia, such as DISC1 mutations, play important roles in astrocyte-mediated regulation of synaptic transmission, neurogenesis, and cognitive processes [413,414]. Hippocampal astrocytes express several serotonin receptor subtypes, including 5-HT1Ars [415], 5-HT2Ars [416], and 5-HT5Ars [417]. Thus, serotonergic signaling may influence glial cell function, further implicating it in the pathophysiology of schizophrenia. Serotonin has also been shown to reduce oxidative stress in the hippocampus [143,162,418], and since oxidative stress and neuroinflammation are established contributors to schizophrenia [419], serotonergic modulation of astrocytes may have neuroprotective and anti-inflammatory roles. Moreover, serotonin’s antioxidant, synaptogenic, and neurogenic effects open promising therapeutic avenues for addressing synaptic loss, impaired plasticity, and neural population deficits in schizophrenia. In this context, physical exercise, which enhances hippocampal neurogenesis via serotonergic pathways [420,421], has been proposed as an adjunctive therapy for improving cognitive and negative symptoms [422,423].

In conclusion, converging lines of evidence highlight the critical role of hippocampal serotonergic signaling in the pathophysiology of schizophrenia. Receptor-level alterations, particularly the upregulation of 5-HT1ARs, and therapeutic effects seen with blockade of 5-HT1ARs, 5-HT3Rs, and 5-HT6Rs, support the view that the hippocampal serotonergic system is a promising target for novel pharmacological interventions. Beyond neurons, the modulation of glial function, redox balance, and neurogenesis by serotonin further emphasizes its multifaceted therapeutic potential. Table 6 presents preclinical evidence supporting the therapeutic potential of serotonergic receptor manipulation, highlighting specific receptor targets and their potential alleviating effects on positive, cognitive, and negative symptoms. Although the serotonergic system appears deeply implicated in hippocampal dysfunction in schizophrenia, several limitations should be noted. Many of the cited studies rely on animal models or postmortem human tissues with inherent limitations in replicability and translational relevance. Moreover, species-specific differences in receptor localization and downstream signaling may constrain generalizability.

### 4.2. Atypical Antipsychotics and the Hippocampus

The primary pharmacological treatment for schizophrenia relies on antipsychotic medications, classified into first-generation (typical) and second-generation (atypical) antipsychotics. Typical antipsychotics act predominantly as high-affinity D2 receptor antagonists or partial agonists [424]. While effective against positive symptoms, they are largely ineffective for negative and cognitive symptoms and are often associated with adverse effects such as extrapyramidal symptoms, psychomotor slowing, and emotional blunting [24]. In contrast, atypical antipsychotics exhibit weaker D2 antagonism and greater affinity for 5-HT2ARs, on which they act as antagonists [221]. These drugs are associated with a lower risk of motor side effects and may offer modest benefits on cognition, though metabolic and cardiovascular complications are frequent [25]. Despite pharmacological advances, negative symptoms and cognitive deficits remain largely refractory to current treatments. Given the role of the serotonergic system in the pathophysiology of schizophrenia and in the mechanism of action of atypical antipsychotics, it presents a promising therapeutic target, particularly in view of its central role in emotional regulation and cognition [425].

Atypical antipsychotics exert multiple effects within the hippocampus, including modulation of neurotransmitter systems, serotonin receptor expression, BDNF levels, and network oscillatory activity. For example, clozapine decreases 5-HT levels in the ventral hippocampus independently of 5-HT1A autoreceptors [20], whereas cariprazine, a D2/D3 partial agonist and 5-HT1AR/5-HT2A/CR antagonist, increases 5-HT levels in this region [426]. Additionally, clozapine and alstonine, but not typical antipsychotics, reduce glutamate uptake in hippocampal slices via D2 and 5-HT2A/CR-related mechanisms, respectively [427], which may be relevant given the role of glutamate hypofunction in hippocampal memory deficits [406]. Drugs like lurasidone and aripiprazole further modulate hippocampal neurotransmission: lurasidone increases dopamine efflux via 5-HT1AR activation and 5-HT7R antagonism, while aripiprazole acts through 5-HT1AR activation [21,428]. Systemic administration of clozapine and olanzapine significantly raises acetylcholine levels in the hippocampus, a process partially mimicked by blockade of 5-HT2A/CRs and 5-HT6Rs [429], and may contribute to improved cognitive function, given the key role of acetylcholine in hippocampal learning and memory.

Importantly, atypical antipsychotics are a heterogeneous drug class. While they share common pharmacodynamic targets, they exhibit varied effects on hippocampal serotonin receptor subtypes, which may contribute to differential therapeutic efficacy or side effect profiles across patients. Given the complexity and heterogeneity of schizophrenia, such receptor-specific actions might represent both opportunities and challenges in optimizing treatment. Atypical antipsychotics, including clozapine and ziprasidone, function as partial agonists at postsynaptic 5-HT1ARs [430]. These drugs can increase hippocampal 5-HT1AR expression, particularly during sensitive developmental periods. For example, acute, but not chronic, administration of aripiprazole increases 5-HT1AR binding in the dorsal hippocampus of female rats, likely through autoreceptor-mediated feedback, which reduces 5-HT efflux [431]. In contrast, chronic clozapine or olanzapine increases 5-HT1AR expression in juvenile but not adult rats, suggesting developmental sensitivity to treatment [432]. Functionally, clozapine also reverses MK-801-induced cognitive deficits via 5-HT1AR partial agonism [433].

Conversely, atypical antipsychotics downregulate 5-HT2AR expression. Clozapine reduces the levels of 5-HT2ARs in human hippocampal cells in vitro [434], while chronic clozapine or olanzapine administration decreases 5-HT2AR density in juvenile rats [432]. Long-term olanzapine treatment also reduces 5-HT2CR binding in dorsal hippocampal CA1 and CA3 regions [435]. Furthermore, in rats with neonatal ventral hippocampal lesions, 5-HT2AR mRNA levels are elevated, and risperidone treatment normalizes these levels [436]. Similarly, adolescent risperidone treatment reverses hippocampal neurogenesis deficits and PV-interneuron loss induced by prenatal infection, likely via 5-HT2A/CR antagonism [437]. Some atypical antipsychotics, including clozapine, olanzapine, and ziprasidone, also exhibit 5-HT6R antagonism [438]. Notably, clozapine, but not the typical antipsychotic haloperidol, reduces 5-HT6R expression in both the dorsal and ventral hippocampus [439]. This is significant, as blockade of 5-HT6Rs elevates dopamine in the ventral, but not dorsal, hippocampus [404,440]. In summary, while more research is needed, current evidence suggests that atypical antipsychotics enhance serotonergic signaling in the hippocampus primarily by increasing 5-HT1AR activity and reducing 5-HT2AR activity. These receptor-level changes may contribute to the cognitive and emotional effects of these drugs and represent potential mechanisms for developing more targeted treatments for schizophrenia.

Brain-derived neurotrophic factor (BDNF) plays a vital role in neuronal development, synaptic plasticity, and survival and is increasingly recognized as a key mediator in the therapeutic effects of atypical antipsychotics. Blockade of 5-HT2ARs, a defining pharmacological feature of atypical antipsychotics, has been linked to elevated hippocampal BDNF levels [441] correlating with improvements in the cognitive and negative symptoms of schizophrenia [442]. Agents such as lurasidone and olanzapine upregulate BDNF expression in the hippocampus [443,444], and olanzapine reverses BDNF suppression induced by blockade of NMDA receptors [445]. However, co-treatment with nicotine occludes olanzapine’s BDNF-enhancing effect, highlighting complexities relevant to smoking comorbidity in schizophrenia [446]. Stress-related reductions in BDNF are also reversed by atypical antipsychotics like quetiapine, especially when co-administered with antidepressants such as venlafaxine [447,448]. Notably, BDNF upregulation by quetiapine requires a hypoglutamatergic state, emphasizing the influence of the neurochemical environment [449]. In contrast, the typical antipsychotic haloperidol consistently decreases BDNF levels [441,450], perhaps reflecting its limited efficacy on negative and cognitive symptoms. The effects of clozapine and risperidone on BDNF are variable and dose-dependent, suggesting additional complexities [444]. Overall, atypical antipsychotics tend to support hippocampal BDNF expression, likely contributing to their therapeutic benefits beyond psychosis. These BDNF-mediated mechanisms offer a promising target for optimizing treatment, potentially via synergistic modulation of serotonergic, glutamatergic, and neurotrophic pathways.

Disruption of hippocampal neural rhythms is increasingly recognized as a core feature in the pathophysiology of schizophrenia. Pharmacological models using NMDA receptor antagonists, such as phencyclidine (PCP), have demonstrated that administration of PCP induces hippocampal desynchronization and disrupts connectivity between the hippocampus and prefrontal cortex (PFC) [451]. Specifically, PCP enhances delta oscillations while suppressing theta rhythms and theta–gamma coupling, patterns associated with impaired information processing and cognitive dysfunction. Notably, atypical antipsychotics such as clozapine and risperidone, but not the typical antipsychotic haloperidol, have been shown to reduce hyperactivity within the PFC–hippocampal circuit. These effects are thought to be mediated by 5-HT1AR activation and 5-HT2AR blockade. However, these drugs do not fully reverse the PCP-induced alterations in hippocampal oscillatory dynamics [451].

**Figure 3:**
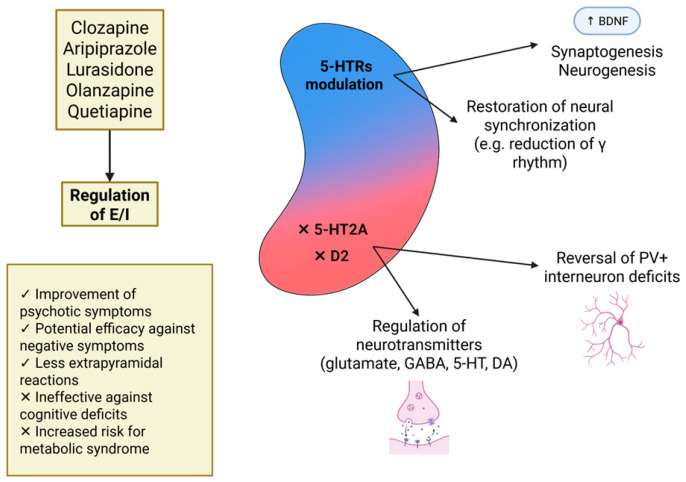
Serotonergic mechanisms of atypical antipsychotic action in dorsal and ventral hippocampus.

In acute hippocampal slice preparations, clozapine has been shown to reduce acetylcholine-induced gamma oscillations, whereas 5-HT3R stimulation enhances gamma activity, possibly via activation of CCK-containing basket cells [452]. These findings are particularly relevant given that positive symptoms in schizophrenia are associated with increased gamma activity, while negative symptoms correspond to reduced gamma oscillations [453,454]. Additional electrophysiological studies reveal that risperidone reduces hippocampal spiking, suppresses theta and gamma oscillations, and decreases theta phase synchronization between the hippocampus and PFC. At the same time, it enhances delta activity, particularly in the dorsal hippocampus [8]. These observations suggest that modulation of hippocampal rhythmic activity contributes to the therapeutic effects of antipsychotics. Although more research is needed to clarify the distinct roles of theta, gamma, and delta oscillations, as well as the involvement of specific 5-HTR subtypes, current evidence supports the notion that restoration of aberrant neural synchrony is a key mechanism underlying the efficacy of atypical antipsychotics in treating schizophrenia. Figure 3 summarizes the main clinical effects and hippocampal molecular mechanisms of atypical antipsychotics, highlighting their impact on neurotransmitter systems, synaptic function, and circuit-level modulation relevant to schizophrenia.

### 4.3. Psychosis of Epilepsy and the Hippocampus

Psychosis and epilepsy are closely related phenomena with overlapping clinical and neurobiological features [8]. Patients with schizophrenia exhibit an increased risk for seizure activity [455], and conversely, antipsychotic medications may lower the seizure threshold, facilitating the onset of epileptic episodes [456]. On the other hand, individuals with epilepsy can develop psychotic symptoms across various clinical presentations, including ictal, postictal, chronic interictal psychosis, forced normalization, and surgery-induced psychosis [457].

Interictal psychosis refers to psychiatric syndromes in epilepsy that arise independently of seizure events and persist for at least 24 h during full consciousness [458]. Subtypes include chronic epileptic psychosis, drug-induced psychosis, and forced normalization, where psychotic symptoms paradoxically emerge concurrently with amelioration of epileptic activity, often accompanied by EEG normalization [459]. Chronic psychosis is associated with long-standing epilepsy and features affective and positive symptoms [457]. Antiepileptic drug-induced psychosis has been linked to factors like female sex, temporal lobe epilepsy, and levetiracetam use, while carbamazepine appears protective [460,461]. These clinical overlaps suggest shared pathophysiological mechanisms, including hippocampal and limbic circuit dysfunction resembling schizophrenia [462,463,464]. Ventral hippocampal hyperactivation and neurodevelopmental anomalies, such as ventricular enlargement, further support a common neural substrate [465,466]. Understanding these mechanisms may clarify how psychosis can emerge as an atypical manifestation of limbic epilepsy.

The hippocampus is widely recognized as the brain region most susceptible to epileptic activity [467,468,469] and, as discussed earlier, plays a central role in the pathophysiology of schizophrenia. Accordingly, it also appears to be critically involved in the emergence of psychosis associated with epilepsy. Schizophrenia-like psychosis of epilepsy is often linked to seizures originating from limbic structures, particularly the hippocampus [470]. In such patients, hippocampal abnormalities have been reported, including structural damage [471], elevated calbindin-positive neuron density in the CA4 region [472], and reduced hippocampal volume [473]. Notably, temporal lobectomy for drug-resistant epilepsy has, in some cases, resulted in postoperative schizophrenia-like psychosis [474], and surgery-induced forced normalization carries a worse prognosis [475].

Hippocampal sclerosis, featuring loss of CA1 pyramidal neurons and disorganization of granule cells, is a hallmark of temporal lobe epilepsy [476]. Seizure activity also impacts neurogenesis in the dentate gyrus (DG), with studies showing either suppression [477] or enhancement [478] of neuronal proliferation. Interestingly, schizophrenia has been hypothesized to begin with damage to the CA1 region [369], while disorganization of the DG may impair pattern separation, potentially contributing to psychotic experiences [406]. Collectively, these findings suggest that hippocampal dysfunction may underlie chronic interictal psychosis in individuals with epilepsy.

The serotonergic system of the hippocampus also plays a critical role in both epilepsy and schizophrenia. Patients with refractory temporal lobe epilepsy and hippocampal sclerosis show reduced hippocampal serotonin levels [479], while limbic seizures have been shown to increase hippocampal 5-HT efflux [480]. Although it remains unclear whether a hyper- or hyposerotonergic state contributes more strongly to seizure susceptibility, serotonergic dysfunction appears to be a common feature of both conditions. Specifically, hippocampal 5-HT1ARs are consistently reduced in epilepsy [5], while 5-HT2ARs show dual pro- and anticonvulsant effects [481]. In contrast, 5-HT3Rs generally exhibit anticonvulsant properties [482]. Other 5-HTR subtypes are less studied in epilepsy but likely play roles. Antiepileptic drugs also influence serotonin metabolism: several increase extracellular 5-HT [483,484], and vigabatrin increases 5-HIAA levels in CSF, indicating enhanced serotonergic turnover [485]. These biochemical changes are notable, as antiepileptic therapy is associated with forced normalization, and withdrawal from such drugs often coincides with remission of psychotic symptoms, potentially through restoration of neurotransmitter balance.

Age-related changes further complicate this relationship. Aging is associated with marked declines in 5-HT1ARs in cholinergic neurons and in the DG, with smaller reductions observed in the CA1 and CA3 regions [486]. Similarly, 5-HT2AR density in the hippocampus declines with age [487]. Importantly, both 5-HT1ARs and 5-HT2ARs are involved in regulating hippocampal neurogenesis, a process implicated in both seizure susceptibility and psychiatric disorders. These findings support the notion that a dysregulated hippocampal serotonergic system is common to both epilepsy and schizophrenia. In individuals with chronic interictal psychosis, impaired serotonin signaling could serve as a shared vulnerability factor, increasing the risk for both disorders. Conversely, chronic epilepsy, particularly involving the temporal lobe, may lower the threshold for psychosis by disrupting neurotransmitter homeostasis, especially involving serotonergic pathways. Furthermore, serotonergic changes induced by aging, epilepsy, or antiepileptic treatment could destabilize hippocampal circuits and contribute to psychotic manifestations. While other neurotransmitter systems, including glutamatergic, GABAergic, and dopaminergic systems, among others, are also clearly implicated, it is tempting to hypothesize that alterations in neuromodulatory receptors with both anticonvulsant and pro-psychotic properties may, in part, explain phenomena like alternating psychosis in epilepsy. For instance, upregulation of 5-HT1ARs may reduce hippocampal excitability, thereby limiting seizures, but could simultaneously enhance vulnerability to psychosis, given the association of 5-HT1AR overactivation with schizophrenia. Similarly, 5-HT3Rs, despite their anticonvulsant effects, are targeted by antagonists to alleviate psychotic symptoms. If this duality is valid, targeted modulation of the serotonergic system, or related neuromodulatory systems, could provide novel therapeutic strategies that address both epilepsy and its comorbid psychosis.

Converging lines of evidence suggest that the ventral hippocampus may serve as a critical nexus between epilepsy and schizophrenia-like psychosis. Most notably, this region is among the most epileptogenesis-prone areas of the brain, exhibiting high susceptibility to seizures and epileptic discharges [488,489,490,491,492,493]. In addition, the neonatal ventral hippocampal lesion model is one of the most widely used animal models of schizophrenia, replicating many core features of the disorder, including cognitive and affective symptoms [16,494]. Moreover, transplantation of GABAergic interneurons into the ventral hippocampus has been shown to alleviate cognitive and negative symptoms in schizophrenia models [36]. Disruption of serotonergic signaling is also evident: blockade of 5-HT1ARs in the ventral hippocampus dysregulates dopaminergic modulation [390,495], and pharmacological models of schizophrenia show increased 5-HT1AR expression in the ventral CA1 [10]. These findings collectively suggest that anatomical lesions or functional disturbances in the ventral hippocampus could produce dual effects, i.e., suppressing seizure activity while inducing psychotic symptoms, potentially explaining phenomena such as alternating psychosis observed in some epilepsy surgery patients. The ventral hippocampus’s role in emotional regulation [496] may also underlie the prevalence of affective symptoms in chronic interictal psychosis. Taken together, these observations underscore the ventral hippocampus as a therapeutic target that requires a cautious, individualized risk–benefit evaluation in the context of epilepsy surgery.

Based on the evidence presented above, we propose three mechanistic pathways for interictal psychosis syndromes, including chronic epileptic psychosis and forced normalization: (1) anatomical or functional damage to the ventral hippocampus, compromising its role in emotional regulation and network homeostasis; (2) chronic epileptic disruption of CA1 and dentate gyrus (DG) microcircuits, impairing pattern separation and contributing to psychosis; and (3) altered neuromodulatory signaling within the hippocampus, particularly serotonergic dysfunction, induced by aging, antiepileptic medications, or seizure activity.

## 5. The Role of Serotonin in Schizophrenia: A Dorsoventral Hippocampal Perspective

The hippocampus, a key structure in emotional regulation, stress responses, episodic memory, and working memory, is central to the pathophysiology of schizophrenia, and impairment in these domains may contribute to disease vulnerability in predisposed individuals [497]. In this final section, we integrate current knowledge on the physiological and pathophysiological roles of the dorsal and ventral hippocampus in the context of schizophrenia. While serotonin is central to this discussion, it is important to acknowledge that schizophrenia is a multifactorial disorder involving multiple neurotransmitter systems and diverse brain regions. In this respect, genetic predisposition [498], neuroinflammatory processes [371], and environmental stressors [374] can disrupt serotonergic signaling. Given the crucial role that serotonin plays in neurodevelopment, such disruptions may impair the development and maturation of hippocampal circuitry, rendering it vulnerable to dysfunction later in life.

Serotonin, by modulating both principal pyramidal neurons and interneurons through its diverse receptor subtypes, plays a pivotal role in regulating hippocampal excitability. Maintaining a physiological E/I balance is essential for normal hippocampal circuit function. Serotonin enhances neuronal excitability by acting on 5-HT2ARs [218,219], 5-HT4Rs [239], and 5-HT7Rs [263], while it reduces excitability through 5-HT3Rs [228,233] and 5-HT6Rs [257,259]. Additionally, certain 5-HTRs exhibit concentration-dependent effects, as observed with 5-HT2AR [217] and 5-HT4R [243]. These actions may reflect the widespread localization of these receptors, including axonal terminals of other neuromodulatory systems, dendrites, and somatic compartments. Consequently, the serotonergic system of the hippocampus plays a multidimensional role across the clinical symptomatology of schizophrenia. It contributes to psychosis via modulation of hippocampal excitability and dopamine transmission, to cognitive symptoms through its effects on plasticity, oscillatory activity, and cholinergic signaling, and to negative symptoms through its involvement in emotional processing, neurotrophic support, and neurogenesis.

**Figure 4:**
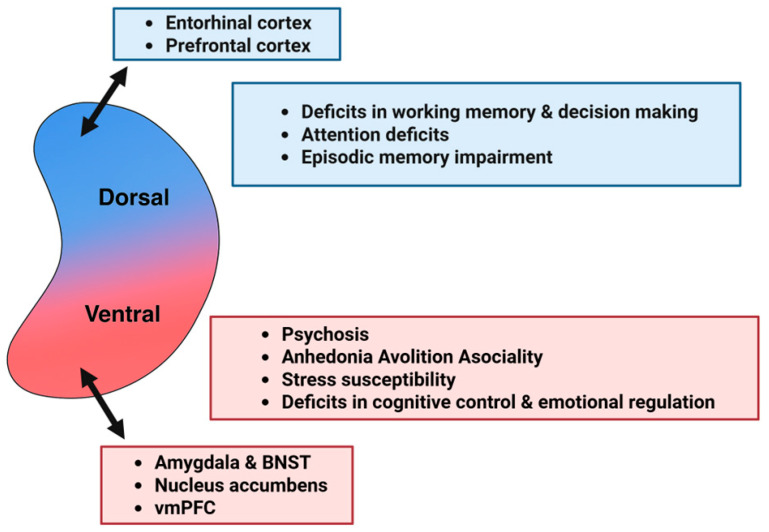
Dorsoventral distribution of schizophrenia-associated impairments in the hippocampus and supporting connectivity profiles.

Interestingly, existing evidence suggests that the dorsal and ventral parts of the hippocampus are involved in different aspects of schizophrenia through alterations in their distinct serotonergic regulation. Schizophrenia is classically characterized by three broad symptom categories: positive symptoms (e.g., psychosis), negative symptoms (e.g., anhedonia, apathy, reduced social engagement), and cognitive symptoms (e.g., deficits in episodic and working memory). Each of these domains is thought to be linked to distinct yet interacting neurobiological mechanisms, in which the hippocampal serotonergic system plays a key modulatory role. The main connections of the dorsal and ventral hippocampus to the positive symptoms, negative symptoms, and cognitive symptoms are summarized in Figure 4. Emerging evidence from neuroimaging studies in clinical populations, as well as preclinical studies in animal models for schizophrenia, indicates that the ventral/anterior hippocampus is implicated in psychosis and emotional dysregulation [34,499], while the dorsal/posterior hippocampus is primarily involved in episodic memory impairments and deficits in working memory [35,500].

### 5.1. Positive Symptoms

Studies using animal models of schizophrenia suggest that serotonergic dysregulation in both the ventral and dorsal hippocampus plays a role in the expression of positive symptoms [379,395]. Moreover, positive symptoms are strongly associated with hyperdopaminergic activity in the mesolimbic pathway, driven in part by increased excitability of the ventral hippocampus and its regulation of mesolimbic dopamine pathways. Specifically, overactivity in the ventral hippocampus increases dopamine release in the nucleus accumbens via projections to the ventral tegmental area, which is linked to hallucinations and delusions [213,501,502,503]. Furthermore, optogenetic stimulation of the ventral subiculum replicates psychosis-related behaviors in rodents, including hyperlocomotion and sensorimotor gating deficits [503]. Therefore, disruption of the serotonergic system of the hippocampus is likely to be implicated in E/I imbalance.

**Figure 5:**
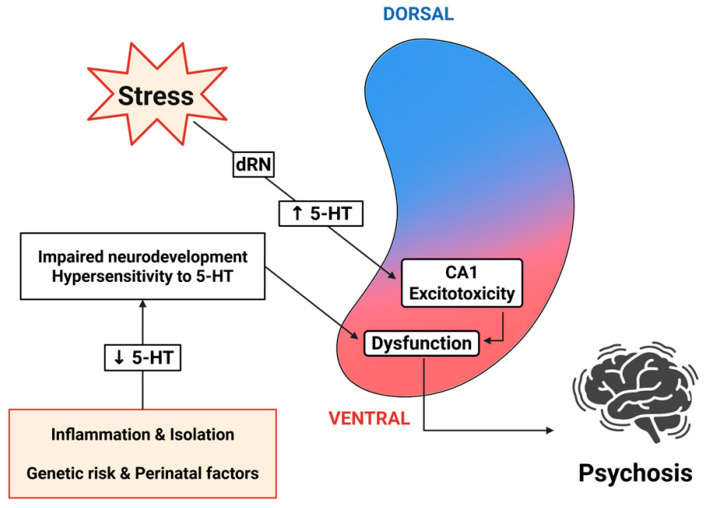
Potential role of disturbed serotonergic modulation in the ventral hippocampus in the development of psychosis.

Reduced baseline serotonin levels in schizophrenia may leave the hippocampus hypersensitive to serotonergic input, contributing to dysregulated network function. Anatomically, the ventral hippocampus receives denser serotonergic innervation from the raphe nuclei compared to the dorsal hippocampus. Moreover, serotonin release in the ventral hippocampus primarily occurs through volume transmission, in contrast to the synapse-specific serotonergic transmission observed in the dorsal hippocampus. In addition, serotonin increases excitability in the ventral hippocampus, whereas it suppresses excitability in the dorsal region via 5-HT1ARs (Figure 1). This distinction may have functional consequences. For instance, stress-induced activation of the dorsal raphe nucleus during sensitive developmental windows may lead to excess serotonin release in the ventral hippocampus, resulting in selective CA1 vulnerability [370]. This evidence is consistent with a proposed mechanism of schizophrenia involving damage to and functional disruption of the CA1 region [369] and provides a rationale for the efficacy of ventral hippocampal lesion models in reproducing schizophrenia-like phenotypes in animals. Figure 5 summarizes the proposed disruptive effects of stress and various environmental risk factors on the ventral hippocampus, which are thought to contribute to the development of psychosis.

The involvement of the serotonergic system in the emergence of positive symptoms is further supported by the fact that serotonergic lesions in the ventral hippocampus result in a mild disruption of PPI in rats, whereas lesions in the dorsal hippocampus exacerbate phencyclidine-induced locomotor hyperactivity and significantly impair PPI [379]. Additionally, local infusion of a 5-HT2CR agonist into the ventral hippocampus rescues MK801-induced PPI deficits [61], while a 5-HT2AR inverse agonist reverses PPI impairments in rats treated with the D1/D2 receptor agonist pergolide [395].

### 5.2. Negative Symptoms

The serotonergic system is a key regulator of emotional processing and motivational behavior, which are impaired in negative symptom domains. Serotonin is involved in the modulation of mood, social drive, and reward sensitivity [504,505]. Hippocampal 5-HTRs mediate anxiety-related behaviors [145] and have been implicated in anhedonia [11] and depressive symptoms [152,189]. In the hippocampus, serotonin also regulates BDNF expression [89,107] and promotes adult neurogenesis [134], both of which are implicated not only in cognitive enhancement but also in emotional resilience and regulation [506].

The ventral hippocampus appears to be also associated with the negative symptoms of schizophrenia. For instance, dysfunction of the ventral hippocampus–nucleus accumbens pathway, which regulates reward processing and motivation, reduces ventral striatal activation during reward anticipation, a neural signature of anhedonia [63,64]. Also, animal models of chronic stress show that hyperactivity in the ventral hippocampus disrupts social interaction and pleasure-seeking behaviors by altering nucleus accumbens dopamine dynamics [63,507], exacerbating negative emotional states associated with psychosis [507]. Further, hyperactivity or lesions of the ventral hippocampus impair social interaction in rodents, mimicking social withdrawal in schizophrenia [508].

Prenatal stress, a recognized risk factor for various psychiatric disorders, has been shown to reduce 5-HT1AR expression in the ventral hippocampus, without affecting receptor levels in the dorsal hippocampus [509]. Notably, the capacity to sustain goal-directed behavior, which is commonly disrupted in schizophrenia [510], is linked to suppression of ventral, but not dorsal, hippocampal activity [511]. This suppression is accompanied by increased activation of the median raphe nuclei, which mediates stimulation of 5-HT3R [511].

### 5.3. Cognitive Deficits

Given the crucial roles of the dorsal hippocampus in cognitive processes, such as attention, working memory, and episodic memory [46,47,48,49], it is likely that it particularly contributes to the emergence of cognitive deficits in schizophrenia. Cognitive deficits in schizophrenia, including impairments in working and episodic memory, have been linked to impaired long-term synaptic plasticity [295], abnormal gamma oscillations [308], and cholinergic dysfunction [381]. Blockade of 5-HT1ARs [139] and 5-HT3Rs [65] in the hippocampus improves working memory in preclinical studies. Combined blockade of 5-HT3Rs and 5-HT6Rs has been shown to reduce gamma oscillations [328], a potentially beneficial effect given the gamma hyperactivity observed in schizophrenia. These receptors (5-HT1ARs, 5-HT3Rs, and 5-HT6Rs) are also involved in modulating LTP, and their activation has been reported to impair its induction [286,290]. As previously mentioned, both hypo- and hyperserotonergic states are implicated in schizophrenia, and it is interesting that enhanced hippocampal serotonergic transmission has been associated with restlessness and reduced cognitive impulsivity [512]. Therefore, blockade of these receptors may help restore LTP, thereby improving cognitive function. Additionally, blocking these receptors has been associated with increased hippocampal acetylcholine release, a mechanism that could further alleviate cognitive impairments. The proposed distinct roles of the two hippocampal subdivisions in schizophrenia, which appear to be accompanied by discrete neuromodulatory actions mediated by serotonin, add to a broader set of dorsoventral differences that span all levels of organization, from genetic/molecular, synaptic, and cellular to network function, connectional, physiological behavior, and pathology; see reviews by [27,28,40,41,42,513,514,515,516,517,518].

### 5.4. Translational Implications

Recent advances in understanding the region-specific roles of 5-HTRs along the dorsoventral axis of the hippocampus open new pathways for targeted therapeutic approaches in schizophrenia. Current antipsychotic treatments act on neurotransmitter systems in a broadly non-selective manner, which limits precise interventions and often leads to common adverse effects [24,25,424,425]. In contrast, the complexity of the serotonergic system and its differential involvement in the dorsal versus ventral hippocampus may enable symptom-specific interventions. For example, blockage of ventral 5-HT2ARs combined with partial agonism of ventral 5-HT1ARs could restore the ventral hippocampal hyperexcitability implicated in psychosis [389]. In parallel, blocking dorsal hippocampal 5-HT3Rs, 5-HT5Rs, 5-HT6Rs, and 5-HT7Rs may improve cognitive deficits in patients with schizophrenia [65,176,253,401]. Interestingly, modulation of these receptors in the ventral hippocampus could also regulate emotional processes, potentially alleviating negative symptoms [177,178,408]. Hence, targeting region-specific 5-HTRs may offer novel therapeutic opportunities for symptom-specific interventions in schizophrenia. Building on the dorsoventral model, several serotonin receptor subtypes represent promising targets for symptom-specific therapeutic strategies in schizophrenia. Finally, another promising direction is targeting serotonergic receptors on astrocytes [415,416,417], which are increasingly recognized as important contributors to the pathophysiology of schizophrenia [412].

## 6. Final Remarks

Although the hippocampal serotonergic system has been extensively investigated, several physiological mechanisms remain incompletely understood, with controversial findings, underexplored domains, and unresolved questions needing further research.

### 6.1. Controversial Findings

As discussed, serotonin exerts complex effects on neuronal physiology and behavior by acting on various receptor subtypes expressed on heterogeneous neuronal populations. Discrepancies are evident between studies using exogenous serotonin application and those investigating endogenous serotonergic transmission, particularly in population spike modulation and long-term synaptic plasticity [4,198,199,273,274,275,276]. Likewise, inconsistencies between in vitro and in vivo studies, such as those concerning 5-HT4R functions in hippocampal circuitry [287,288], suggest a need to account for methodological differences. These divergent findings may be due to dose-dependent receptor effects, variations in experimental paradigms, or developmental and species-specific factors.

Of particular interest are findings in which both agonism and antagonism of the same receptor subtype lead to similar outcomes. For example, both activation and inhibition of hippocampal 5-HT6Rs have been reported to exert procognitive effects [176]. A similar ambiguity surrounds the role of 5-HT2ARs in schizophrenia, where studies report both elevated and reduced expression levels in the hippocampus [388,391,393]. Since many atypical antipsychotics target 5-HT2ARs, it remains unclear whether such alterations reflect intrinsic disease pathology or are confounded by chronic pharmacotherapy. This underlines the need for comparative studies in drug-naive patients and validated animal models. Moreover, inconsistencies in the reported molecular effects of atypical antipsychotics [20,427,431,432] highlight the pharmacological heterogeneity of this drug class, while differences in neurotransmitter levels across agents [426,427,429] may encourage personalized treatment approaches based on receptor and circuit-level profiles.

### 6.2. Limitations, Open Questions, and Future Directions

Our proposed model of a functionally segregated involvement of dorsal and ventral hippocampus in schizophrenia, with a focus on serotonergic modulation, is subject to several limitations. Most available data derive from preclinical animal models, while human postmortem studies are largely correlative and often lack longitudinal resolution. Additionally, the current framework does not integrate other neuromodulatory systems known to regulate hippocampal activity, such as the noradrenergic and cholinergic systems, which may interact with serotonergic signaling in complex, region-specific ways. Given the crucial implications of dorsoventral specialization in hippocampal function, future studies should prioritize direct comparisons between dorsal and ventral (anterior-posterior in humans) hippocampal segments.

Importantly, sex and developmental stage represent important yet underexplored factors in serotonergic signaling and hippocampal function. Evidence indicates that serotonin receptor expression, synaptic plasticity, and hippocampal connectivity vary by sex and across developmental time points [519,520,521]. These differences may influence susceptibility to schizophrenia and response to serotonergic interventions. However, most existing studies do not discriminate findings by sex or developmental stage, limiting generalizability. Future research should prioritize sex- and age-specific analyses to refine mechanistic models and therapeutic strategies. Addressing this gap will be critical for developing more individualized, developmentally informed treatment approaches.

The evidence presented here highlights the need for further rigorous investigation of serotonergic regulation in the dorsal and ventral hippocampus, aiming to link this regulation to the complex manifestations of schizophrenia and potentially other serotonin-related disorders. This underscores the dorsoventral heterogeneity of serotonergic modulation in the hippocampus as a key area for future research and the development of targeted therapeutic approaches. Recent advances in targeted delivery technologies (such as nanoparticle-based drug delivery), optogenetics, and chemogenetics may enable precise modulation of either the dorsal or ventral hippocampus [503,522,523,524,525]. Thus, the dorsoventral 5-HT framework provides a conceptual foundation for developing next-generation therapies that are symptom- and circuit-specific, potentially leading to greater efficacy and fewer adverse effects. Furthermore, structural and functional neuroimaging studies of the hippocampus in patients with schizophrenia [34,35] may help to define anterior-posterior hippocampal endophenotypes, offering potential diagnostic and prognostic biomarkers for the disorder. Advanced imaging techniques such as pharmacological MRI [526,527] hold promise for elucidating serotonergic dynamics in vivo, particularly in patients with schizophrenia. Additionally, exploring techniques such as focused ultrasound or microinfusion devices [528,529,530] for localized hippocampal administration of serotonergic agents in preclinical models could be particularly beneficial. Finally, the development of more complex translational models, such as double- or triple-hit animal paradigms [16], may better replicate the multifactorial nature of psychiatric disorders, advancing our understanding of disease mechanisms and informing the development of targeted interventions across diagnostic boundaries.

### 6.3. Conclusions

Accumulating evidence demonstrates that serotonergic modulation along the hippocampal dorsoventral axis is highly specialized, with the dorsal and ventral segments of the structure differentially regulating cognitive and emotional–behavioral functions, respectively. In schizophrenia, convergent data from animal models and patient studies indicate elevated 5-HT1AR expression and serotonergic hypofunction in the dorsal hippocampus, underlying cognitive impairments, while the ventral hippocampus exhibits hyperactive 5-HT2A/3R signaling and denser serotonergic innervation, associated with psychotic symptoms, negative symptoms, and stress responsivity. This “dorsoventral serotonin imbalance” model is supported by molecular, imaging, and electrophysiological evidence showing that these alterations disrupt E/I balance, neural plasticity, and network dynamics. Clinically, these insights provide an opportunity for the development of region- and receptor-specific therapeutic strategies, using, e.g., receptor-selective agents or advanced delivery techniques to enable more precise, symptom-specific interventions that are undoubtedly needed in schizophrenia. Further research is necessary, but the dorsoventral model proposed here provides a framework for both mechanistic studies and the development of improved therapies.

## Figures and Tables

**Table 1:** Effects of 5-HTRs on serotonin, dopamine, acetylcholine, and noradrenaline in the hippocampus.

	Serotonin	Dopamine	Acetylcholine	Noradrenaline
5-HT1AR	↓ (receptors in raphe nuclei)	↑	↓	↑
5-HT1BR	↓	Unknown	↓	Unknown
5-HT2R	↓	↓	↓, ↑	↓
5-HT3R	↑	Unknown	↑, ↓	↑, ↓
5-HT4R	↑	Unknown	↑	Unknown
5-HT5R	Unknown	Unknown	Unknown	Unknown
5-HT6R	Unknown	↑, ↓	↓	↑
5-HT7R	↑	↑	Unknown	↑

↑ indicates an increase, and ↓ indicates a decrease in neurotransmitter levels.

**Table 2:** Hippocampal 5-HTRs: synopsis of regional localization, cellular expression, molecular targets, and physiological roles.

Receptor	Regional Localization	Cellular Expression	Molecular Targets	Physiological Roles	References
5-HT1A/B	DG, CA3, CA1	PN and IN	G_i/o_, GIRK, Ca^2+^ channels, NMDA, AMPA, MAPK-ERK	Neuroprotection, neurogenesis, emotion, cognition	[74,75,76,77,78,134,135,136,137,138,139,140,141,142]
5-HT2A/C	DG, CA3, CA1	PN and IN	G_q/11_, PLC- PIP3-DAG, K^+^ channels, PLA2-AA, ERK, PDZ, BDNF	Neuroprotection, mood, anxiety, learning, memory	[85,86,87,88,89,90,91,143,144,145,146,147,148,149,150,151]
5-HT3	DG, CA3, CA1	IN	Cation channel (Na^+^, K^+^, Ca^2+^), IGF-1	Neuroprotection, neurogenesis, fear extinction, working memory, learning	[65,92,93,94,95,96,97,152,153,154,155,156,157,158,159]
5-HT4	DG, CA3	PN	Gs, PKA-AC-cAMP, K^+^ channels, PDE, β-catenin, CREB, BDNF, AKT	Neuroprotection, emotion, cognition	[62,98,99,100,101,102,103,104,105,160,161,162,163,164,165,166,167,168,169]
5-HT5	CA1	PN	G_i/o_, GIRK	(No studies in the hippocampus)	[9,106,107,108,109,110,111,112,170,171,172,173,174,175]
5-HT6	DG, CA3, CA1	PN and IN	Gs, PKA-AC-cAMP, ERK1/2, BDNF	Mood, anxiety, memory consolidation	[113,114,115,116,117,118,119,120,121,176,177,178,179,180,181,182,183,184,185]
5-HT7	DG, CA3, CA1	PN	Gs, PKA-AC-cAMP, TrkB, AMPA, NMDA, CREB, 5-HT1A	Neuroprotection, emotional learning, stress regulation, cognition	[79,80,81,82,83,84,111,176,186,187,188,189,190,191,192,193,194,195,196,197]

**Table 3:** 5-HT receptors in dorsal vs. ventral hippocampus and schizophrenia relevance.

Receptor	Dorsal Hippocampus	Ventral Hippocampus	Main Expression	Principal Circuit Actions	Symptoms Linked to Schizophrenia	Therapeutics (Potential/Existing)
5-HT1A	High (CA1, DG); extrasynaptic	Moderate (CA3, DG)	PNs, INs	Inhibitory role in dorsal hippocampus; excitatory role in ventral hippocampus	Cognitive deficits (dorsal hippocampus); positive symptoms (ventral hippocampus)	Antagonists (cognitive rescue); partial agonists
5-HT2A	Moderate	Moderate–High (CA3, CA1)	PNs, INs	↑ Glutamate ↑ GABA	Psychosis, emotional dysregulation	Antagonists (atypical antipsychotics), inverse agonists
5-HT2C	High (Str. oriens/radiatum CA1)	High (CA3)	PNs, INs	Similar to 5-HT2A	Mood/anxiety symptoms, sensorimotor gating	Agonists/antagonists (experimental)
5-HT3	Moderate (INs)	Moderate	INs; PNs (in humans)	Fast excitation of INs	Working memory, negative symptoms	Antagonists (ondansetron, tropisetron)
5-HT4	Moderate (CA3, DG)	Moderate	PNs	Excitatory ↑ LTP, ↑ ACh	Cognitive, emotional, neurogenic roles	Agonists/antagonists (early clinical stage)
5-HT5A/B	Low–Moderate (DG, CA1, CA3)	Low–Moderate	PNs, INs	Unknown/potentially inhibitory	Cognition, social behavior	Blockers (preclinical, memory/social rescue)
5-HT6	Moderate (DG, CA1, INs)	Moderate	PNs, CCK+ INs	Inhibitory; modulate LTP	Memory, mood, negative symptoms	Antagonists (SB-742457, clinical trials)
5-HT7	CA3 > CA1 > DG	Moderate	PNs	Excitatory	Cognitive flexibility, stress	Agonists/antagonists (experimental)

↑ indicates an increase or enhancement.

**Table 4:** Effects of 5-HTRs on long-term synaptic plasticity, hippocampal oscillations, and network excitability.

	LTP/LTD	Theta Rhythm	Gamma Rhythm	SWRs	E/I
5-HT1R	↓ CA1 [7] ↑ DG [280]	↓ [8]	↓ [8,314,324]	↑ sharp waves [348] ↓ ripples [347]	↓ dorsal hippocampus ↑ ventral hippocampus
5-HT2R	↑ CA1 [281,282]	↑ 5-HT2A, [8] ↓ 5-HT2C [315]	↑ [325] ↑ 5-HT2A [8]	↓ sharp waves [348]	↑
5-HT3R	↓ CA1, [283,284] ↓ CA3 [285,286]	↓ [283,284,316]	↓ [316,326,327] ↑ 5-HT3 + 5-HT6 [328]	↓ ripples [347] no effect on sharp waves [348]	↓
5-HT4R	↑ DG, ↓ CA3 [244]	↑ [324]	unknown	no effect on sharp waves [348]	↑
5-HT5R		unknown	unknown	unknown	unknown
5-HT6R	↓ CA1 [290] ↑ DG [289]	↓ [318,319]	↓ [319] ↑ 5-HT3 + 5-HT6 [328]	unknown	↓
5-HT7R	↑ CA1 [291] ↑ DG [292]	unknown	unknown	unknown	↑

The arrows in the E/I column are based on interpretations derived from the literature analysis.

**Table 5:** Summary of alterations in hippocampal serotonergic markers in animal models and postmortem human studies in schizophrenia.

	Finding	Condition	Relevance to Schizophrenia	References
5-HT levels	Reduced	Neuroinflammation, social isolation, STOP mutation, hyperdopaminergic state	Risk factors	[371,372,373,374]
TPH2	Increased	Maternal inflammation	Risk factors	[376]
	Decreased	STOP mutation		[373]
5-HT1A	Increased	Isolation rearing, prenatal infection, MK-801 administration, postmortem human studies	Translational importance	[10,374,388,393]
5-HT1B	Increased	Postmortem human studies	Human findings	[388]
5-HT2A	Increased	Postmortem human studies	Human findings	[388]
	Decreased			[384,393]
5-HT4	Reduced	Animal model for anhedonia	Negative symptoms	[11]
5-HT6	Reduced	Postmortem human studies	Human findings	[403]

**Table 6:** Therapeutic effects of 5-HTR manipulation in preclinical schizophrenia models.

Receptor Manipulation	Therapeutic Effect	Relevance to Schizophrenia	References
5-HT1A blockage	Restores PPI, hyperlocomotion, working memory performance	Positive and cognitive symptoms	[137,138,389]
5-HT2A blockage/partial agonism	Restoration of frontoseptohippocampal circuit activity, reverses PPI deficits	Positive and cognitive symptoms	[394,395]
5-HT3 blockage	Antipsychotic actions, improvement of learning and working memory	Positive and cognitive symptoms	[65,153,399,401]
5-HT5 modulators	Procognitive effects, emotional regulation	Cognitive and negative symptoms	[253]
5-HT6 blockage	Procognitive, anxiolytic, antidepressant, and antiepileptic effects	Cognitive and negative symptoms	[113,176,177,178,184,185,408,409]
5-HT7 blockage	Procognitive effects	Cognitive symptoms	[411]

## Abbreviations

The following abbreviations are used in this manuscript:

5-HT	5-Hydroxytryptamine
5-HTR	5-Hydroxytryptamine Receptor
AC	Adenyl Cyclase
AMPA	α-Amino-3-hydroxy-5-methyl-4-isoxazolepropionic Acid
BDNF	Brain-Derived Neurotrophic Factor
BNST	Bed Nucleus of the Stria Terminalis
dRNu	Dorsal Raphe Nucleus
CA1/CA3	Cornu Ammonis Regions 1 and 3
CB1	Cannabinoid Receptor Type 1
CCK	Cholecystokinin
CREB	cAMP Response Element-Binding Protein
DAG	Diacylglycerol
DG	Dentate Gyrus
DH	Dorsal Hippocampus
E/I	Excitation/Inhibition
EPSP	Excitatory Postsynaptic Potential
ERK	Extracellular Signal-Activated Protein Kinase
fMRI	Functional Magnetic Resonance Imaging
GABA	Gamma-Aminobutyric Acid
GIRK	G-Protein-Activated Inwardly Rectifying Potassium Channel
GPCR	G-Protein-Coupled Receptor
Ih	Hyperpolarization-Activated Current
IN	Interneuron
LTD	Long-Term Depression
LTP	Long-Term Potentiation
MAPK	Mitogen-Activated Protein Kinase
MDMA	3,4-Methylenedioxymethamphetamine
MK	MK-801 (NMDA Receptor Antagonist)
mPFC	Medial Prefrontal Cortex
mRNu	Median Raphe Nucleus
NMDA	N-Methyl-D-Aspartate
NVHL	Neonatal Ventral Hippocampal Lesion
PCA	Para-Chloroamphetamine
PDE	Phosphodiesterase
PIP 3	Phosphatidylinositol (3,4,5)-triphosphate
PKA	Protein Kinase A
PLC	Phospholipase C
PN	Pyramidal Neuron
PPI	Prepulse Inhibition
PV	Parvalbumin
SERT	Serotonin Transporter
SSRIs	Selective Serotonin Reuptake Inhibitors
SST	Somatostatin
SWRs	Sharp Waves–Ripples
TrkB	Tropomyosin Receptor Kinase B Receptor
VH	Ventral Hippocampus
